# Interpretable machine learning-based predictive model for malnutrition in subacute post-stroke patients: an internal and external validation study

**DOI:** 10.3389/fnut.2025.1692020

**Published:** 2026-01-05

**Authors:** Ping Sun, Junqi Luan, Guotao Duan, Qingqing Sun, Genli Liu

**Affiliations:** 1 Second Clinical Medical College, Heilongjiang University of Chinese Medicine, Harbin, Heilongjiang Province, China; 2 Second Department of Rehabilitation, The First Affiliated Hospital of Heilongjiang University of Chinese Medicine, Harbin, Heilongjiang Province, China; 3 First Department of Pediatrics, The First Affiliated Hospital of Heilongjiang University of Chinese Medicine, Harbin, Heilongjiang Province, China; 4 Fourth Department of Acupuncture and Moxibustion, The Second Affiliated Hospital of Heilongjiang University of Chinese Medicine, Harbin, Heilongjiang Province, China

**Keywords:** CAT, machine learning, multicenter study, predictive model, risk factors, subacute stroke

## Abstract

**Background:**

Malnutrition is a critical concern associated with increased mortality rates and adverse outcomes in stroke adults undergoing subacute rehabilitation. Despite its clinical significance, predictive tools for assessing malnutrition risk in this population remain limited. This study aimed to develop and validate an interpretable machine learning (ML) model to predict malnutrition risk among stroke patients during subacute rehabilitation.

**Methods:**

This multicenter study comprised a development cohort of 802 patients from a single institution, which randomly split into training and testing sets at a 7:3 ratio. An external validation cohort of 345 patients was recruited from an independent hospital. Feature selection was conducted using the Least Absolute Shrinkage and Selection Operator (LASSO) regression combined with the Boruta algorithm. Eight ML models—Logistic Regression (LR), Random Forests (RF), Extreme Gradient Boosting (XGBoost), Light Gradient Boosting Machine (LGBM), Support Vector Machines (SVM), k-Nearest Neighbors (KNN), Neural Network (NNet), and CatBoost (CAT)—were trained utilizing five-fold cross-validation. These models were evaluated using metrics such as discrimination, calibration curve, and decision curve analysis (DCA). Model interpretability was assessed via Shapley Additive Explanations (SHAP) analysis.

**Results:**

The CAT algorithm exhibited superior predictive model in the training and testing sets, achieving an area under the receiver operating characteristic curve (AUC) of 0.848 (95% CI: 0.817–0.879) and 0.806 (95% CI = 0.752–0.861), respectively. Calibration metrics underscored the model’s robustness and DCA emphasized its clinical utility. External validation further corroborated the generalizability of the CAT model, demonstrating an AUC of 0.772; (95% CI: 0.723–0.820). SHAP analysis identified age, handgrip strength, and Barthel Index (BI) score as the most significant predictors of malnutrition.

**Conclusion:**

This study successfully developed and validated an ML model for efficiently screening malnutrition risk in patients with subacute stroke. The interpretable CAT-based model serves as a clinically actionable tool, enabling early stratification of malnutrition risk in subacute stroke patients. This facilitates the implementation of targeted nutritional interventions and personalized rehabilitation strategies, potentially improving outcomes in this vulnerable population.

## Introduction

Stroke remains a leading cause of mortality and long-term disability worldwide, placing significant socioeconomic burdens on healthcare systems and communities (1). Post-stroke malnutrition, a prevalent yet frequent overlooked complication, affects 19 to 72% of patients upon admission to rehabilitation (2). This condition is strongly associated with poor functional recovery, an increased risk of stroke-associated pneumonia, prolonged hospitalization, higher mortality rates, and elevated healthcare costs (3). The subacute phase of stroke recovery, spanning from onset to 3 months post-stroke, represents a critical therapeutic window during which neuroplasticity peaks and targeted interventions can maximize functional recovery (4). Paradoxically, this period is also marked by increased vulnerability to malnutrition due to complications, metabolic dysregulation, and impaired self-care capacity (5). Post-stroke malnutrition results from a variety of etiological pathways. Dysphagia, affecting up to 50% of stroke survivors, directly impairs nutritional intake and is a primary cause of malnutrition (6). Furthermore, poor functional status—manifested by hemiparesis, reduced mobility, depression, post-stroke dementia, or systemic inflammation—severely disrupts nutritional balance (7). Malnutrition also exacerbates sarcopenia, a progressive decline in muscle mass and strength that impairs rehabilitation effectiveness and functional outcomes (8). Despite its clinical significance, malnutrition in subacute stroke patients remains systematically underrecognized, primarily due to the absence of standardized assessment protocols (9).

Identifying malnutrition is crucial for implementing effective nutritional interventions during the subacute phase of rehabilitation recovery (4). However, current nutritional assessment methods exhibit significant limitations, primarily due to the absence of a universally accepted definition of malnutrition and the lack of a gold standard for evaluating nutritional status (10). Although the Global Leadership Initiative on Malnutrition (GLIM) criteria recently established a global consensus on diagnostic criteria for malnutrition in adults (11), they provide standardized phenotypic and etiologic diagnostic criteria that are impractical for rapid assessment in acute and subacute stroke settings and fail to proactively assess malnutrition risk (12). Furthermore, the dynamic nature of post-stroke recovery, during which the critical subacute rehabilitation window coincides with peak malnutrition risk, necessitates tools that offer early, actionable insights (2, 13). Machine learning (ML), a subset of artificial intelligence, focuses on developing algorithms that autonomously improve their performance through experience (14). Over the past few decades, ML has demonstrated proficiency in handling large-scale datasets and has been widely applied in medicine for early detection, diagnosis, and outcome prediction (15). Recently, a growing body of studies has emerged on ML techniques for predicting cerebrovascular events, particularly in stratifying stroke patients to optimize therapeutic interventions and forecast outcomes (16).

Several studies have developed and validated diagnostic models utilizing ML algorithms to predict malnutrition across diverse cohorts (17–19). However, limited research has specifically addressed malnutrition prediction in subacute stroke patients. To fill these gaps, this study proposes an interpretable ML-based predictive model specifically designed for malnutrition risk stratification in subacute post-stroke patients. By integrating variables such as dysphagia, sarcopenia indicators, and functional status metrics, the model seeks to balance predictive performance with interpretability, enabling clinicians to identify high-risk patients and customize interventions during this critical rehabilitation phase. This approach facilitates the implementation of nutrition-focused rehabilitation protocols and addresses the need for rapid-assessment tools across various clinical settings.

## Methods

### Study design and population

**Figure 1:**
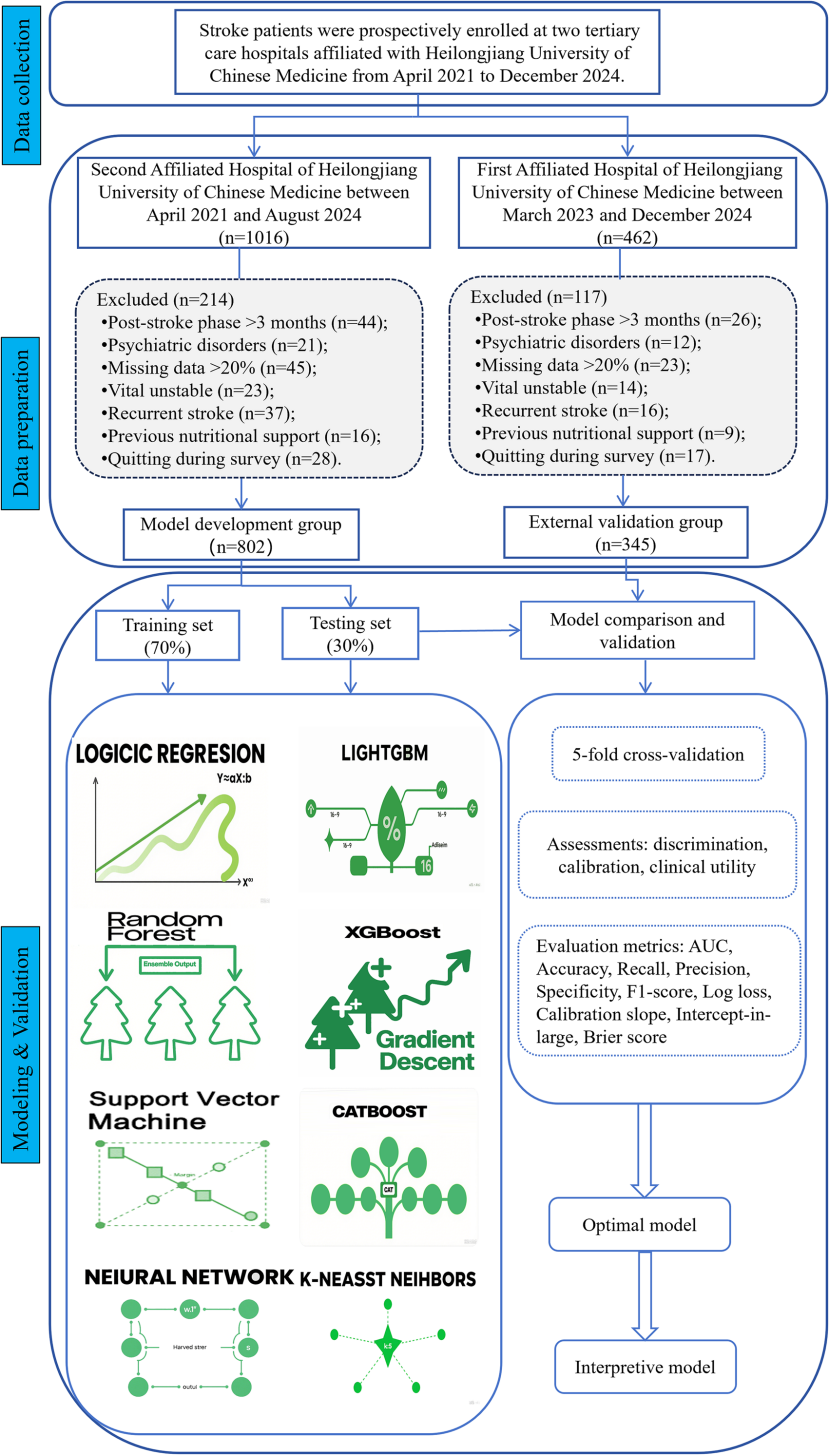
Flow diagram of the study.

This prospective, multicenter, cross-sectional study consecutively enrolled stroke patients admitted to inpatient rehabilitation departments at two tertiary care hospitals affiliated with Heilongjiang University of Chinese Medicine from April 2021 to December 2024. The model development cohort consisted of patients recruited from the Second Affiliated Hospital between April 2021 and August 2024 (39-month recruitment period), whereas the external validation cohort comprised participants from the First Affiliated Hospital enrolled between March 2023 and December 2024 (22-month recruitment period). Inclusion criteria were: (1) age ≥18 years; (2) first- diagnosis of ever ischemic or hemorrhagic stroke, confirmed via magnetic resonance imaging (MRI) or computed tomography (CT); (3) subacute phase (1–3 months after stroke onset); (4) sufficient cognitive function and language ability to permit assessment completion; (5) voluntarily participated in the study and provided written informed consent; (6) stable vital signs; (7) availability of comprehensive nutritional status evaluations. Exclusive criteria included: (1) diagnosis of transient ischemic attack or subarachnoid hemorrhage; (2) history of prior stroke; (3) active psychiatric disorders; (4) concurrent severe life-threatening diseases (e.g., malignant tumors, end-stage cardiac/renal dysfunction); (5) missing data > 20%; (6) receipt of nutritional supplementation within 3 months prior to admission; (7) estimated life expectancy <6 months. The recruitment process is illustrated in Figure 1.

This study adhered to the ethical principles of the Declaration of Helsinki and received approval from the respective institutional review boards: the Second Affiliated Hospital (Approval No. [2021]K179) and the Frist Affiliated Hospital (Approval No. KY[2023]983) of Heilongjiang University of Chinese Medicine. Written informed consent was obtained from all participants.

### Malnutrition screening and diagnosis

This study employed a two-step methodology to identify malnutrition in subacute post-stroke patients, following the GLIM criteria (20). The same outcome definitions and diagnostic protocols were applied uniformly to both cohorts to ensure consistency. Initially, nutritional risk stratification was conducted within 24 h of admission using the Nutritional Risk Screening 2002 (NRS-2002) (21). This validated tool assessed three domains: nutritional status impairment, disease severity, and age-adjusted risk stratification. Patients scoring ≥3 points were classified as nutritionally at-risk and progressed to secondary assessment. The subsequent malnutrition diagnosis followed GLIM standards, requiring fulfillment of at least one phenotypic and one etiological criterion. The phenotypic criteria included: (1) weight loss: > 5% within 6 months or > 10% beyond 6 months; (2) low BMI: BMI < 18.5 kg/m^2^ for patients aged < 70 years or < 20 kg/m^2^ for those ≥ 70 years; (3) reduced muscle mass, measured via calf-circumference (< 34 cm for males and < 33 cm for females) (22) or handgrip strength (< 28 kg for males or < 18 kg for females) (10). The etiological criteria included (1) reduced nutrient intake: persistent consumption ≤50% of estimated energy requirements for > 7 days or sustained intake reduction of any magnitude for ≥14 days; (2) inflammatory: acute or chronic inflammatory burden resulting from stroke-related complications, comorbidities, or systemic disease (23).

### Data collection

Demographic and clinical data were systematically gathered upon hospital admission through electronic health records (EHRs) and questionnaires. Demographic characteristics included age (in years), gender, body mass index (BMI, kg/m^2^), medical payment method; education level, employment status, living status, monthly household income, drinking history, smoking history, comorbidities (hypertension, diabetes, dyslipidemia, cardiovascular disease, digestive disease, chronic kidney disease), eating habits, polypharmacy status (concurrent use of ≥5 medications), functional independence measured by Barthel Index (BI) score, and handgrip strength (assessed via dynamometer on the non-hemiparetic side). Clinical stroke profiles included days since stroke onset, stroke classification (ischemic/hemorrhagic), lesion localization, stroke severity quantified using the National Institutes of Health Stroke Scale (NIHSS), and post-stroke complications (including pneumonia, dominant arm paresis, anorexia, dysphagia, dysarthria/aphasia). Biological parameters were analyzed from fasting venous blood samples obtained within 24 h of admission: hemoglobin (HGB, g/L), total protein (TP, g/L), albumin (Alb, g/L), total cholesterol (TC, mmol/L), triglycerides (TG, mmol/L), serum creatinine (Scr, mmol/L), fibrinogen (FIB. g/L), D-dimer (μg/mL), high-density lipoprotein cholesterol (HDL-C, mmol/L), low-density lipoprotein cholesterol (LDL-C, mmol/L), C-reaction protein (CRP, mg/L), uric acid (UA, μmol/L), white blood cell count (WBC, 10^9^/L), neutrophils (NEU, 10^9^/L), lymphocytes (LYM, 10^9^/L), platelets (PLA, 10^9^/L), neutrophil-lymphocyte ratio (NLR), platelet-lymphocyte ratio (PLR), and prognostic nutritional index (PNI, calculated as Alb + 5 × LYM).

### Data preprocessing

Initially, variables (e.g., laboratory indicators) across participating centers were standardized to ensure consistency and robustness in multicenter data integration. Variables with >20% missing values were excluded from the analysis to reduce bias associated with incomplete data. Remaining missing values were imputed using a random forest-based approach, which utilizes feature correlations to iteratively predict plausible replacements while maintaining data structure (24). The procedure ran for a maximum of 10 iterations, with convergence automatically assessed through stabilization of the out-of-bag (OOB) imputation error (relative change tolerance: <0.5% between iterations). Continuous variables were imputed via regression forests, while categorical variables were imputed via classification forests (Supplementary Figure S1). Subsequently, continuous variables were normalized through Z-score transformation. Categorical variables (e.g., gender, comorbidities) were converted into dummy variables using one-hot encoding, ensuring compatibility with ML algorithms while avoiding ordinal assumptions.

### Feature selection process

To balance model parsimony with robust feature identification, we employed a hybrid two-step feature selection process that included the Least Absolute Shrinkage and Selection Operator (LASSO) and Boruta algorithms to identify independent risk factors for malnutrition. LASSO regression, a regularization technique, was utilized to mitigate overfitting while identifying parsimonious predictors through L1-penalized optimization (25). The optimal regulation parameter (*λ*) was determined using 10-fold cross-validation with one standard error (λ.1se) as the selection criterion. Concurrently, the Boruta algorithm iteratively assessed variable importance against permuted “shadow features,” retaining only attributes that demonstrated statistically significant predictive power (26). This approach was chosen to leverage their complementary strengths: LASSO addresses multicollinearity through coefficient shrinkage, while Boruta’s permutation-based ensemble method captures non-linear and interaction effects often overlooked by linear models (27, 28).

### Model development

Eight ML algorithms were employed to develop the malnutrition prediction framework: Logistic Regression (LR), Random Forests (RF), Extreme Gradient Boosting (XGBoost), Light Gradient Boosting Machine (LGBM), Support Vector Machines (SVM), k-Nearest Neighbors (KNN), Neural Network (NNet), and CatBoost (CAT). A detailed summary of the algorithmic rationale is provided in Supplementary Table S1. In this study, the dataset from the model development cohort was randomly divided into training and testing sets in a 7:3 ratio. To address potential overfitting and optimize the predictive models, the final hyperparameters for each model were obtained through five-fold cross-validation combing with grid search (Supplementary Table S2).

### Model validation

The predictive performance of the optimal model was rigorously evaluated using both internal and external validation cohorts, employing multiple evaluation metrics, including the area under the receiver operating characteristic curve (AUC) with 95% confidence intervals (CIs), accuracy, precision, recall, specificity, Brier score, log loss, and F1 score. Calibration accuracy was evaluated by comparing observed versus predicted probabilities via calibration curves, while clinical utility was quantified through decision curve analysis (DCA) to estimate net benefit across probability thresholds. Statistical differences in AUC values between candidate models were examined using the Delong test. The final model was selected following a comprehensive comparative analysis of predictive performance, calibration integrity, and clinical applicability.

### Model interpretability

Model interpretability was rigorously assessed using the Shapley Additive Explanation (SHAP) framework (29), a method derived from game theory that calculates feature importance through coalitional contribution analysis. SHAP values yield mathematically consistent attribution scores, decomposing each prediction into the marginal impact of individual feature while preserving the global model behavior. To operationalize this framework, we implemented two complementary analytical visualizations: (1) a SHAP beeswarm plot, which quantitatively illustrates the impact of features at the population level by aggregating SHAP values across the entire cohort, with each point representing the magnitude of a feature’s directional contribution (positive/negative) for a single observation; (2) individualized waterfall plots were constructed to illustrate how specific features cumulatively modify baseline population risk estimates, ultimately producing final predictions for representative clinical cases.

### Statistical analysis

All statistical analyses were conducted using SPSS Statistics (version 27.0; IBM Corp.) and R software (version 4.4.3; R Foundation for Statistical Computing). Continuous variables characterized by a non-normal distribution were presented as medians with interquartile ranges (IQRs). Inter-group differences in medians were evaluated using the Mann–Whitney U test. Categorical variables were expressed as frequencies and percentages and were subjected to comparisons using the chi-squired test. A two tailed *p* value < 0.05 was considered statistically significant.

## Results

### Baseline characteristics

A total of 802 eligible stroke patients were included in the model development cohort. The median age of the enrolled patients was 64 years (range: 44–85), with 71.4% (573/802) being male. Ischemic stroke was the most prevalent subtype, accounting for 86.5% of cases. Baseline characteristics of the patients are summarized in Table 1. The overall prevalence of malnutrition within the cohort was 57.2% (459/802). Statistical analysis showed significant differences in age (*p* < 0.001), BMI (*p* = 0.030), smoking history (*p* = 0.048), eating habits (*p* < 0.001), BI score (*p* < 0.001), handgrip strength (*p* < 0.001), NIHSS score (*p* < 0.001), loss of appetite (*p* = 0.032), dysphagia (*p* < 0.001), FIB (*p* = 0.006), and CRP (*p* = 0.025) between the malnutrition and non-malnutrition groups (Supplementary Table S3). For model development, the cohort was randomly divided into training (*n* = 562) and testing (*n* = 240) groups. Demographic and clinical variables exhibited comparable distributions between the two groups, with no significant differences observed (*p* > 0.05, Supplementary Table S4).

**Table 1:** Comparison of model performance in the training and testing cohorts.

Model	Accuracy (95% CI)	AUC (95% CI)	Recall (95% CI)	Precision (95% CI)	F1 score (95% CI)	Specificity (95% CI)	Log loss (95% CI)	Brier score (95% CI)
Training set								
LR	0.696 (0.660–0.733)	0.769 (0.730–0.807)	0.787 (0.743–0.831)	0.704 (0.653–0.749)	0.743 (0.705–0.778)	0.581 (0.520–0.640)	0.568 (0.532–0.602)	0.193 (0.178–0.208)
RF	0.738 (0.699–0.776)	0.798 (0.761–0.835)	0.834 (0.792–0.874)	0.734 (0.688–0.780)	0.781 (0.745–0.814)	0.617 (0.555–0.678)	0.537 (0.495–0.583)	0.180 (0.163–0.198)
XGBoost	0.733 (0.692–0.770)	0.821 (0.787–0.855)	0.793 (0.748–0.836)	0.746 (0.700–0.791)	0.769 (0.731–0.803)	0.657 (0.594–0.719)	0.511 (0.468–0.553)	0.171 (0.154–0.189)
LGBM	0.674 (0.639–0.712)	0.726 (0.685–0.768)	0.787 (0.740–0.831)	0.680 (0.637–0.730)	0.730 (0.696–0.765)	0.532 (0.470–0.594)	0.609 (0.582–0.633)	0.210 (0.198–0.221)
CAT	0.762 (0.724–0.795)	0.848 (0.817–0.879)	0.790 (0.742–0.836)	0.785 (0.739–0.832)	0.787 (0.751–0.820)	0.726 (0.667–0.779)	0.489 (0.462–0.520)	0.162 (0.150–0.176)
SVM	0.699 (0.664–0.738)	0.766 (0.726–0.805)	0.755 (0.709–0.801)	0.720 (0.677–0.766)	0.737 (0.700–0.776)	0.629 (0.567–0.688)	0.573 (0.538–0.609)	0.195 (0.180–0.210)
NNet	0.719 (0.681–0.756)	0.796 (0.760–0.833)	0.796 (0.752–0.839)	0.727 (0.674–0.772)	0.760 (0.724–0.792)	0.621 (0.556–0.678)	0.540 (0.507–0.575)	0.182 (0.168–0.197)
KNN	0.699 (0.662–0.737)	0.763 (0.723–0.802)	0.818 (0.776–0.858)	0.696 (0.648–0.745)	0.753 (0.715–0.788)	0.548 (0.482–0.611)	0.578 (0.546–0.609)	0.197 (0.183–0.211)
Testing set								
LR	0.725 (0.671–0.779)	0.775 (0.716–0.834)	0.759 (0.690–0.830)	0.780 (0.711–0.846)	0.769 (0.714–0.821)	0.674 (0.582–0.765)	0.555 (0.507–0.601)	0.189 (0.168–0.208)
RF	0.708 (0.650–0.767)	0.756 (0.694–0.818)	0.814 (0.753–0.874)	0.733 (0.667–0.800)	0.771 (0.715–0.822)	0.547 (0.453–0.646)	0.584 (0.515–0.647)	0.197 (0.170–0.223)
XGBoost	0.725 (0.679–0.787)	0.783 (0.725–0.841)	0.786 (0.738–0.861)	0.765 (0.698–0.833)	0.776 (0.734–0.832)	0.632 (0.526–0.727)	0.555 (0.494–0.615)	0.187 (0.162–0.212)
LGBM	0.671 (0.612–0.733)	0.732 (0.668–0.797)	0.759 (0.686–0.824)	0.714 (0.641–0.782)	0.736 (0.676–0.789)	0.537 (0.442–0.635)	0.600 (0.559–0.644)	0.206 (0.188–0.226)
CAT	0.738 (0.683–0.792)	0.806 (0.752–0.861)	0.766 (0.695–0.828)	0.793 (0.725–0.857)	0.779 (0.723–0.830)	0.695 (0.605–0.785)	0.539 (0.494–0.582)	0.182 (0.161–0.201)
SVM	0.700 (0.637–0.762)	0.774 (0.715–0.832)	0.731 (0.654–0.800)	0.763 (0.684–0.830)	0.746 (0.685–0.800)	0.653 (0.557–0.745)	0.571 (0.516–0.628)	0.193 (0.169–0.217)
NNet	0.721 (0.667–0.775)	0.772 (0.714–0.831)	0.766 (0.696–0.839)	0.771 (0.709–0.836)	0.768 (0.716–0.816)	0.653 (0.553–0.747)	0.561 (0.510–0.612)	0.191 (0.168–0.213)
KNN	0.717 (0.658–0.771)	0.715 (0.648–0.782)	0.800 (0.736–0.863)	0.748 (0.677–0.812)	0.773 (0.716–0.822)	0.589 (0.490–0.689)	0.618 (0.564–0.676)	0.212 (0.189–0.238)

AUC, the area under the receiver operating characteristic ROC curve; LR, Logistic Regression; RF, Random Forests; XGBoost, Extreme Gradient Boosting; LGBM, Light Gradient Boosting Machine; SVM, Support Vector Machines; KNN, k-Nearest Neighbors; NNet, Neural Network; CAT, CatBoost.

### Feature selection

**Figure 2:**
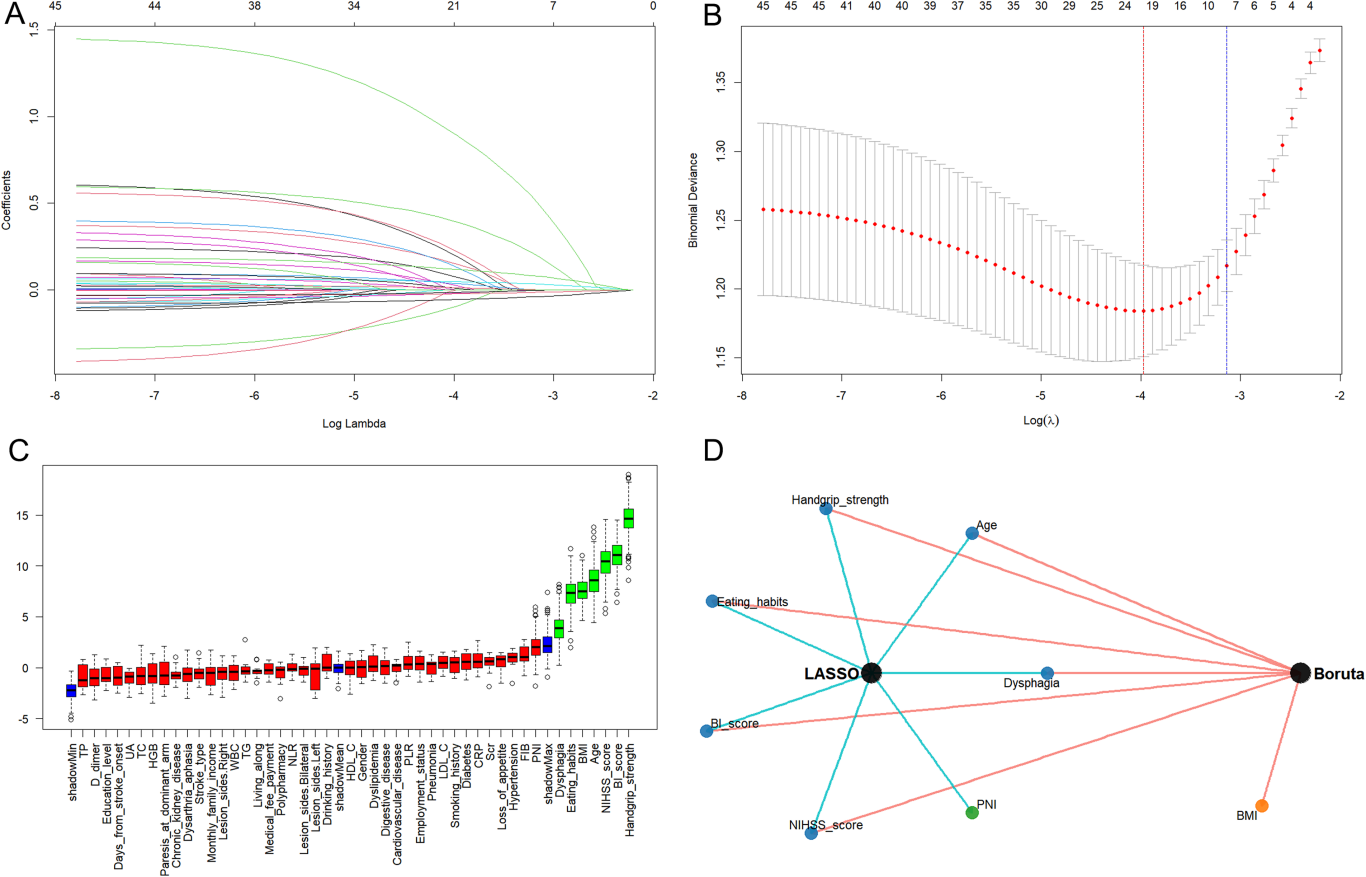
Feature screening process. **(A)** Dynamic plot for LASSO variable selection. **(B)** Predictor screening using LASSO model with 10-fold cross-validation. The left dashed line indicates the *λ* value corresponding to the minimum error (λ.min), while the right dashed line represents the λ value within one standard error (λ.1se). **(C)** Feature identification using Boruta algorithm. The bule boxes represent the minimum, average, and maximum shadow score. Green boxes indicate important variables, while red ones are rejected. **(D)** Common predictors identified by both LASSO and Boruta.

The LASSO regression method was utilized to analyze independent variables associated with malnutrition (Figure 2A). The optimal regularization parameter (*λ*) was determined using 10-fold cross-validation, yielding a λ.1se value of 0.0434 (Figure 2B). This process identified seven candidate variables that were predictive of malnutrition: age, BI score, dysphagia, eating habits, handgrip strength, NIHSS score, and PNI. Subsequently, the Boruta algorithm was applied, independently selecting seven critical predictors: age, BI score, dysphagia, eating habits, handgrip strength, NIHSS score, and BMI (Figure 2C). A comparative analysis of feature subsets derived from LASSO and Boruta revealed a consensus set of six variables shared by both methods: age, BI score, dysphagia, eating habits, handgrip strength, and NIHSS score (Figure 2D).

### Model development and performance evaluation

**Figure 3:**
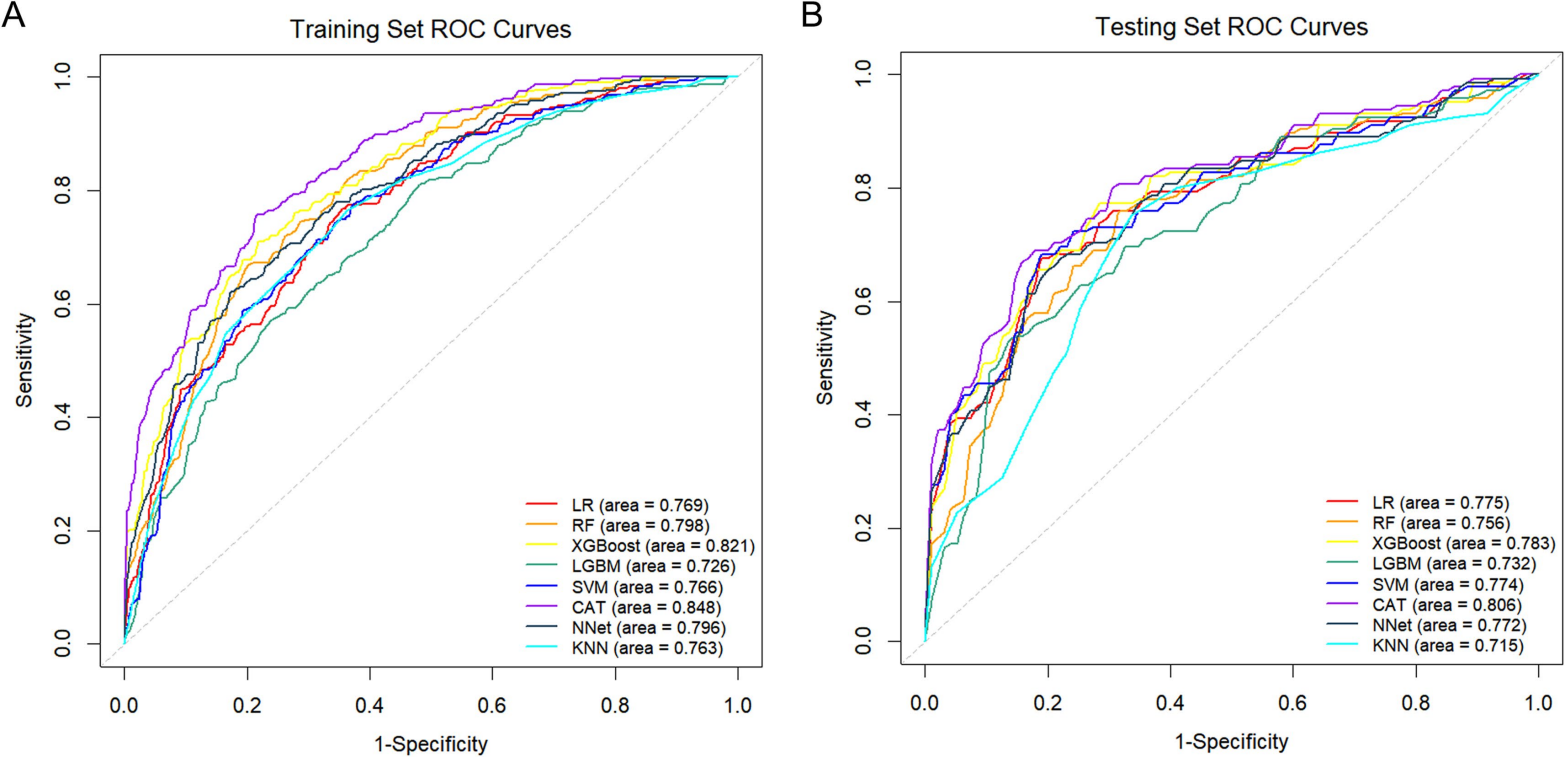
Evaluation of the discriminative ability of the eight ML models. **(A,B)** ROC curves for the training and testing sets.

**Figure 4:**
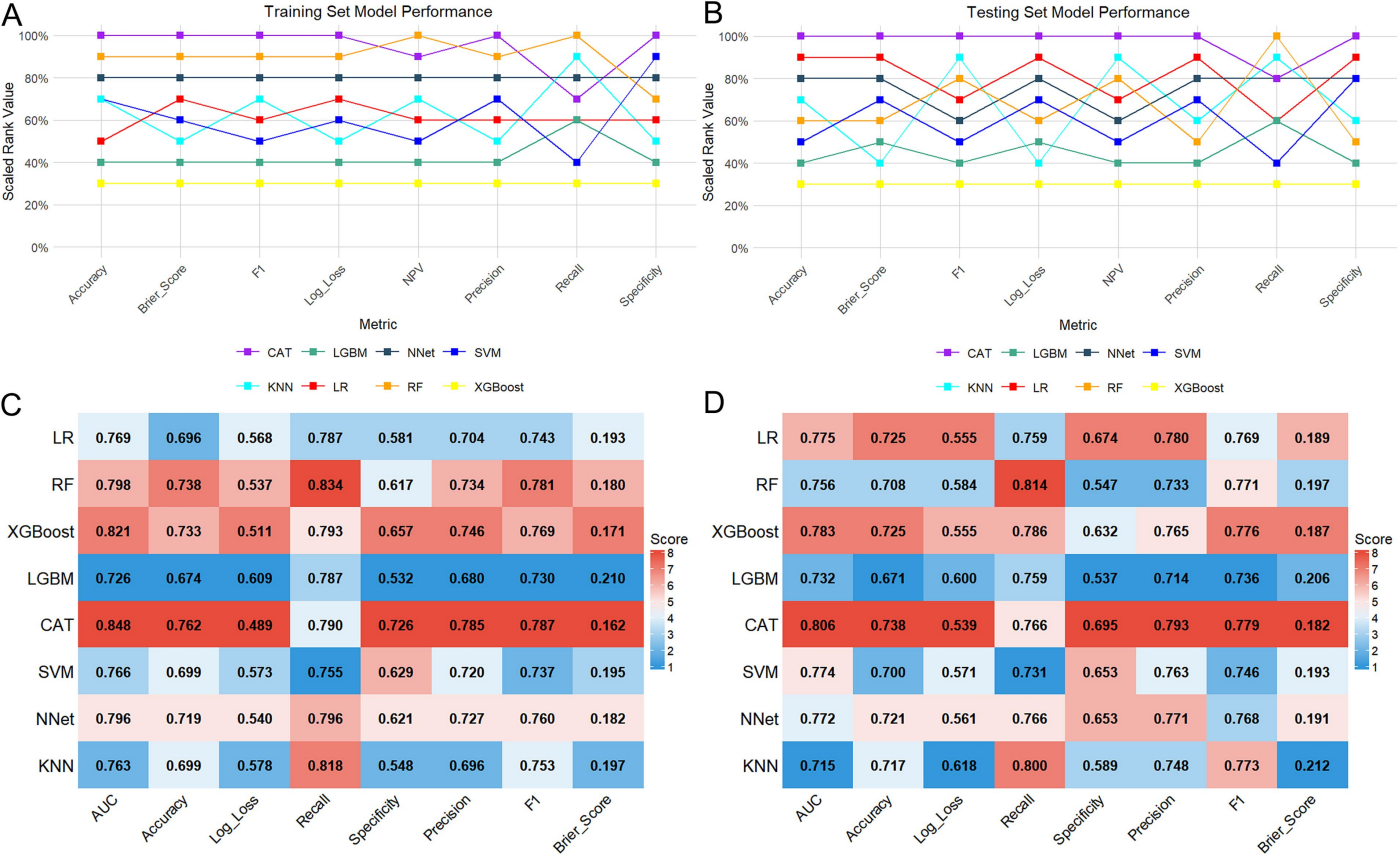
Comparison of the performance of eight ML models. **(A,B)** Line plots comparing the evaluation metrics across eight ML models in the training and testing cohorts. **(C,D)** Heatmap of evaluation metrics for eight ML models in the training and testing cohorts.

Eight ML algorithms, including LR, RF, XGBoost, CAT, LGBM, KNN, NNet, and SVM were employed to develop and optimize malnutrition prediction models. In the training set, the CAT model demonstrated the best overall performance, achieving the highest AUC of 0.848 (95% CI: 0.817–0.879). This was followed by XGBoost (AUC = 0.821; 95% CI: 0.787–0.855) and RF (AUC = 0.798; 95% CI: 0.761–0.835; Figure 3A). Furthermore, the CAT model exhibited superior performance across multiple metrics, including accuracy (0.762), precision (0.785), specificity (0.726), F1 score (0.787), and log loss (0.489; Figures 4A,C; Table 1). In the testing set, the CAT model maintained the highest AUC at 0.806 (95% CI: 0.752–0.861), followed by XGBoost (AUC = 0.783; 95% CI: 0.725–0.841) and LR (AUC = 0.775; 95% CI: 0.716–0.834) models (Figure 3B). Consistently, the CAT model outperformed all other evaluated models, yielding the highest accuracy (0.738), precision (0.793), specificity (0.695), F1 score (0.779), and log loss (0.539; Figures 4B,D; Table 1).

**Figure 5:**
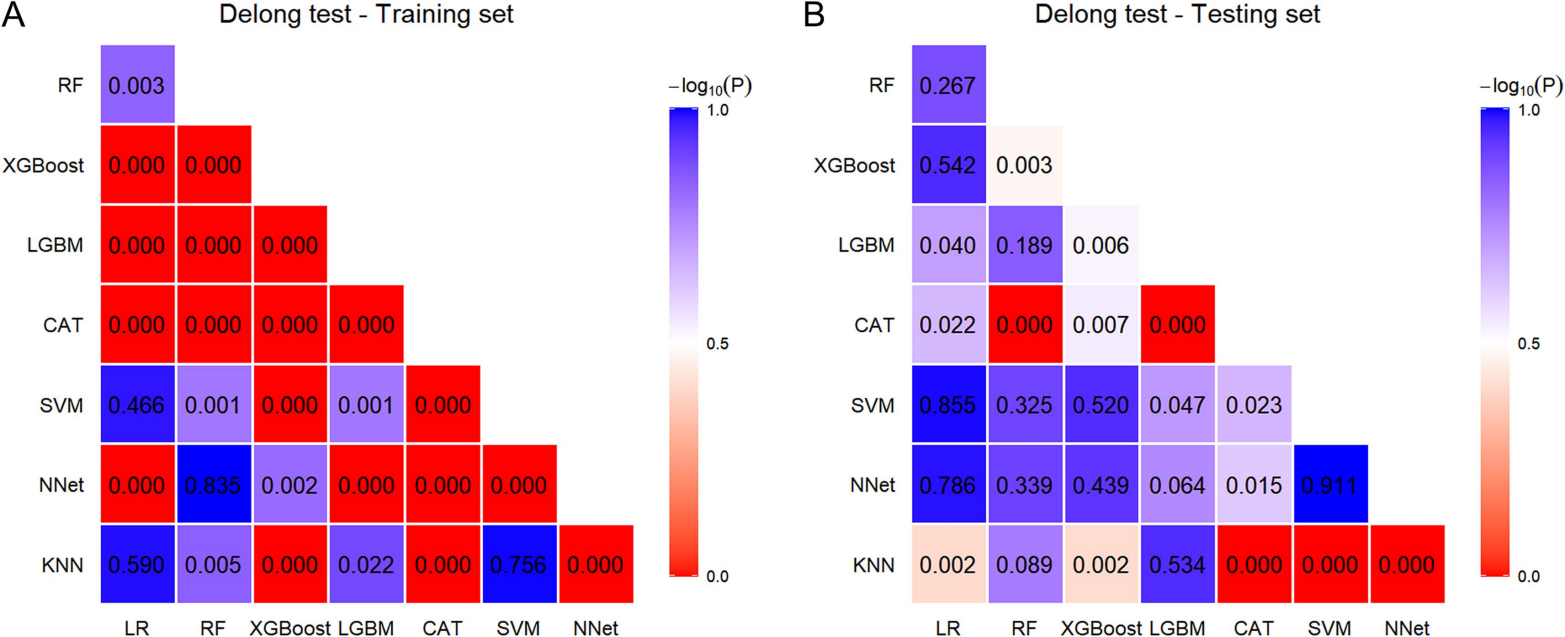
Comparison of AUC for eight ML models using the Delong test. **(A)** Comparison within the training set. **(B)** Comparison within the testing set.

In the training set, the Delong test confirmed that the AUC of the CAT model differed significantly from that of the other models (all *p* < 0.05; Figure 5A; Supplementary Table S5). Similarly, in the testing set, the CAT model yielded a significantly higher AUC compared to the other models (Figure 5B; Supplementary Table S5).

### Cross-validation and model stability

To assess the stability of the CAT model, five-fold cross-validation was conducted on the training dataset. Across the five folds, the model yielded AUC values ranging from 0.721 (95% CI: 0.619–0.823) to 0.827 (95% CI: 0.750–0.904), resulting in a mean AUC of 0.763 (95% CI: 0.706–0.820; Supplementary Figure S2; Supplementary Table S6). The minimal variation of the CAT model throughout the cross-validated iterations underscored its reliability.

### Calibration curves and decision curve analysis

**Figure 6:**
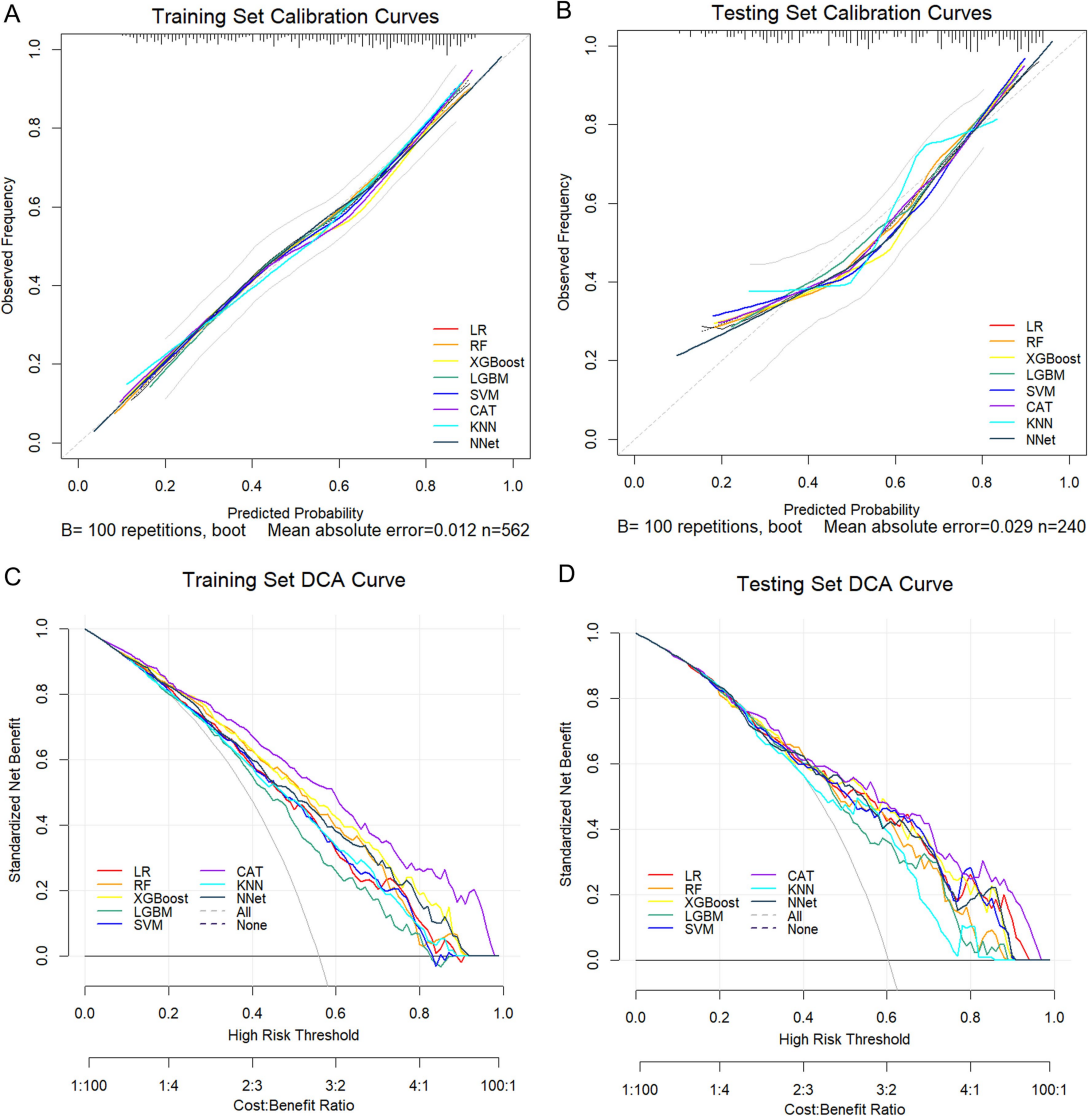
Calibration curves and clinical utility of the eight ML models. **(A,B)** Calibration curves for the training and testing sets. **(C,D)** DCA curves for the training and testing sets.

In the training set, calibration analysis demonstrated strong agreement between predicted and observed malnutrition probabilities across most models. The CAT model exhibited near-ideal calibration, with a Brier score of 0.162 (Figure 6A; Table 1). DCA showed that the CAT model performed the best across entire threshold range, followed by XGBoost and NNet models (Figure 6C). In the testing set, calibration curves showed that the CAT model demonstrated excellent agreement between predicted and observed malnutrition probabilities, with the lowest Brier score of 0.182 (Figure 6B; Table 1). DCA further indicated that the CAT model offered the greatest net benefits across a wide range of threshold probabilities, consistently outperforming other models (Figure 6D). Collectively, based on the comprehensive evaluation, the CAT algorithm emerged as the optimal model for predicting malnutrition in subacute post-stroke patients.

### External validation

**Figure 7:**
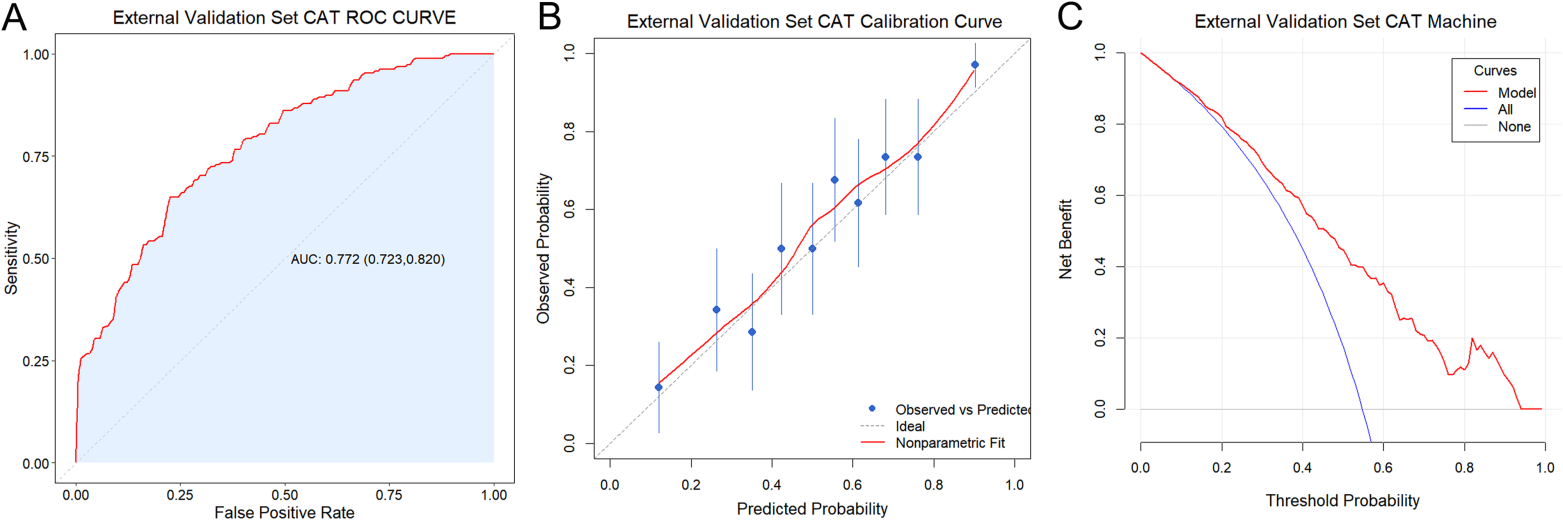
External validation of the CAT-based predictive model. **(A)** ROC curve. **(B)** Calibration curve. **(C)** DCA.

External validation of the CAT model was performed using a prospective cohort (*n* = 345) from an independent hospital. The baseline characteristics of this cohort were summarized in Supplementary Table S7. Although the AUC of the CAT model decreased slightly in the external validation (AUC = 0.772; 95% CI: 0.723–0.820), it still had the best discriminative capacity (Figure 7A; Supplementary Table S8). Calibration analysis revealed strong agreement between predicted and observed malnutrition probabilities (Figure 7B), characterized by a Brier score of 0.193 (Supplementary Table S8). DCA underscored the model’s clinical applicability, demonstrating sustained net benefit across a wide range of threshold probabilities (Figure 7C). These findings further underscored the CAT model’s strong stability and reliability in predicting malnutrition risk across different datasets.

### SHAP for model interpretation

**Figure 8:**
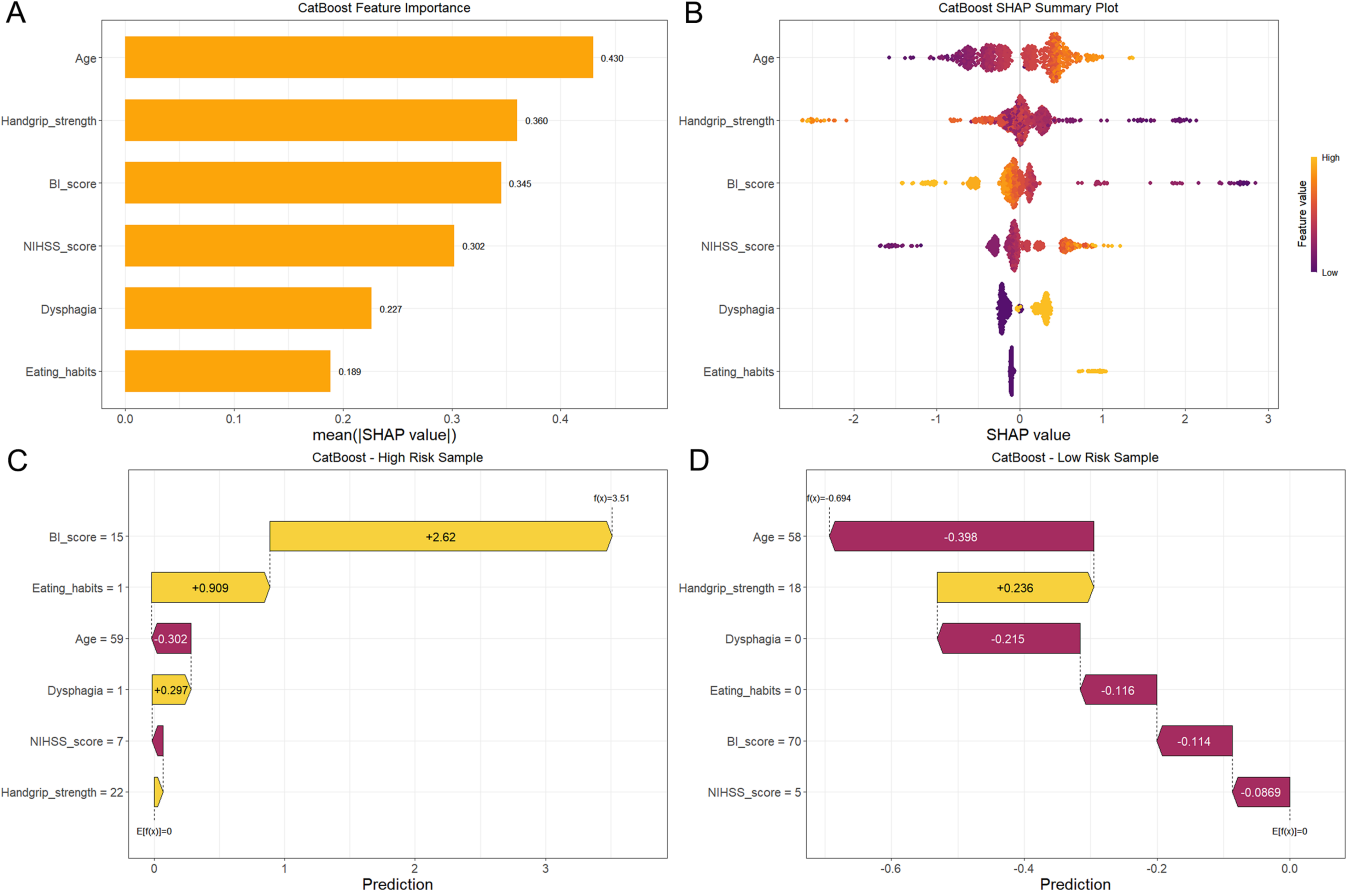
Visual interpretation of CAT-based predictive model by SHAP. **(A)** SHAP summary plot. **(B)** SHAP beeswarm plot. Yellow color represents a high feature value for malnutrition, whereas purple represents a low feature value. **(C)** SHAP waterfall plot for a case of malnutrition. **(D)** SHAP waterfall plot for a case of non-malnutrition. Yellow indicates positive contributions and red indicates negative impacts.

The SHAP framework was employed to interpret the contribution of predictor variables to malnutrition risk predictions generated by the CAT model. The SHAP summary plot quantified feature importance based on mean absolute SHAP values, ranking predictors in descending order of influence: age, handgrip strength, BI score, NIHSS score, dysphagia, and eating habits (Figure 8A). A complementary beeswarm plot further elucidated the directional relationships between individual features and predicted outcomes (Figure 8B). In this visualization, SHAP values (horizontal axis) represent the magnitude and direction of feature effects, with color intensity denoting high (yellow) or low (purple) feature magnitudes. Features positioned farther from the neutral SHAP values of zero exhibited stronger associations with malnutrition risk. Specifically, advanced age, increased NIHSS score, reduced handgrip strength, lower BI score, presence of dysphagia, and tube feeding dependency contributed to malnutrition outcomes.

Case-specific interpretations were generated using SHAP waterfall plots to illustrate individual prediction mechanisms (Figures 8C,D). In these visualizations, yellow arrows indicate features contributing to higher risk (positive SHAP values), whereas red arrows denote protective effects (negative SHAP values). The baseline model output, denoted as E[f(x)], represented the expected risk across the population, whereas f(x) reflected the model’s predicted output for a specific individual. For the malnourished patient (Figure 8C), the model yielded a prediction value of 3.51, substantially exceeding the baseline value of 0. This elevated risk was primarily attributed to three factors: unfavorable BI score (+2.62), tube feeding (+0.909), and the presence of dysphagia (+0.297). Conversely, for the non-malnourished patient (Figure 8D), the prediction value was −0.694, markedly below the baseline. Protective factors contributing to this risk reduction included younger age (−0.398), absence of dysphagia (−0.215), oral feeding (−0.116), a higher BI score (−0.114), and a lower NIHSS score (−0.0869).

## Discussion

This study conducted a comprehensive analysis of clinical data from 802 subacute post-stroke patients in China to develop and validate a ML-based predictive model for malnutrition risk. To the best of our knowledge, this represents the first interpretable ML framework specifically designed for malnutrition screening in stroke populations. The CAT algorithm was identified as the optimal model following a rigorous comparative evaluation of predictive performance across multiple ML algorithms. This model demonstrated strong predictive performance across the training, testing, and external validation cohorts. To ensure clinical interpretability, SHAP analysis was employed to elucidate the model’s decision-making logic. The SHAP analysis identified six key predictors of malnutrition: age, NIHSS score, handgrip strength, BI score, dysphagia, and eating habits. Regarding clinical translation, future work should prioritize integrating this model into EHR systems via user-friendly digital interfaces. Such integration, augmented by real-time, patient-specific explanations of risk factors, is essential to foster clinician trust and streamline the model’s adoption into routine care protocols.

Previous studies have developed predictive models to assess malnutrition risk in stroke populations. For example, nomograms validated in diverse stroke cohorts have demonstrated satisfactory predictive accuracy (30, 31). However, existing models predominantly focus on acute-phase stroke patients and rely on specialized assessments, potentially limiting their clinical scalability. Furthermore, conventional multivariate regression approaches are susceptible to small-sample bias and often lack generalizability when modeling complex, nonlinear relationships among variables. To address these limitations, ML algorithms that leverage routinely collected clinical data offer a promising avenue for enhancing predictive performance. Recent literature highlights the superiority of ML models over traditional LR for stratifying malnutrition risk across diverse clinical populations (17, 19, 32). Consistent with these advancements, in our study, the CAT model outperformed conventional LR in discriminative accuracy across training, testing, and external validation cohorts. These findings underscore the potential of ML-based tools to facilitate the early identification of high-risk individuals in clinical practice, thereby alleviating the burden on healthcare systems through targeted interventions.

Unlike linear classifiers such as LR, which rely on predefined parametric assumptions, the CAT model inherently addresses feature importance estimation through an iterative, hierarchical splitting mechanism. This process systematically partitions heterogeneous clinical data into interpretable decision pathways while mitigating overfitting through ensemble techniques (33). This adaptability is particularly advantageous for malnutrition prediction, given that multifactorial risk factors (e.g., dysphagia severity, functional status) exhibit nonlinear interdependencies that conventional linear models often fail to capture adequately (19). Performance evaluations demonstrated that the CAT-based model consistently outperformed competing algorithms across multiple aspects—including discrimination, calibration, and clinical utility—in both internal and external validations. These findings highlight the utility of the CAT framework for malnutrition risk stratification in subacute post-stroke care. In contrast to traditional nutritional screening tools such as GLIM and NRS-2002, which are susceptible to variability in patient-report data and evaluator-dependent biases, the CAT model addresses these limitations by leveraging ML to provide an objective, data-driven evaluation framework. This approach minimizes subjectivity and facilitates timely nutritional interventions in clinical settings.

ML models are frequently criticized for their inherent “black-box” nature, which limits insight into their decision-making processes. To enhance the interpretability of our CAT algorithm, we employed the SHAP framework to elucidate the model’s predictive mechanisms (34). Although traditional logistical regression identified associations between predictors and outcomes, it is constrained by assumptions of linearity and often lacks the granularity required to capture complex feature interactions. In contrast, our SHAP analysis not only validated established predictors—such as age, dysphagia, and handgrip strength—but also quantified the nuanced contributors of additional variables, including NIHSS score, dietary patterns, and BI score. Crucially, SHAP revealed potential non-linear relationships and complex interactions that were overlooked by logistic regression, thereby deepening the understanding of the mechanisms underlying malnutrition in subacute post-stroke patients (35). These insights facilitate individualized risk stratification, providing clinicians with a comprehensive perspective on how various clinical, biochemical, and demographic factors synergistically influence malnutrition risk. For instance, while logistic regression quantified age as an independent linear predictor, SHAP elucidated its dynamic interaction with comorbidities (e.g., elevated NIHSS scores) doubling risk multiplicatively. By translating complex model outputs into actionable evidence, this approach enhances both predictive accuracy and clinical utility, bridging the divide between algorithmic complexity and clinical applicability.

The interplay of older age, dysphagia, and tube feeding dependency emerged as a critical nexus influencing malnutrition risk in our cohort. Advanced age inherently predisposes patients to nutritional deficits due to age-related physiological changes—including diminished physiological function reserve, altered appetite regulation, and reduced metabolic efficiency—which are often compounded by various comorbidities (36). These vulnerabilities are exacerbated in post-stroke contexts, where older adults frequently experience delayed recovery of independent swallowing function (37). Dysphagia, a prevalent sequela of stroke, directly disrupts oral intake and elevates aspiration risk, necessitating compensatory strategies such as texture-modified diets or tube feeding (38). However, while tube feeding ensures caloric delivery in dysphagic patients, delayed initiation or suboptimal calibration of enteral formulas—common in resource-constrained settings—may fail to meet elevated protein-energy demands, particularly in older adults with reduced physiological resilience (39). These observations align with prior studies (9, 40), demonstrating a significant association between older age, dysphagia and malnutrition. Furthermore, previous literature has consistently identified tube feeding as a significant predictor of malnutrition risk among post-stroke patients (41, 42). Consequently, these findings underscore the importance of regular nutritional monitoring, tailored formular adjustments in tube-fed cohorts, and early dysphagia rehabilitation to mitigate long-term dependency.

The association of elevated NIHSS scores and diminished BI scores with malnutrition underscores the complex interaction between stroke severity, functional dependency, and nutritional compromise. Higher NIHSS scores, reflecting greater neurological impairment, are associated with prolonged immobilization, systemic inflammation, and metabolic dysregulation (43, 44). Concurrently, diminished BI scores, indicative of reduced functional independence, exacerbate malnutrition risk by limiting patients’ ability to self-feed or access adequate nutrition, particularly in settings with insufficient caregiver support (45). This functional dependency may delay or disrupt meal schedules, reduce dietary diversity, and precipitate unintentional weight loss (30, 46). Furthermore, reduced handgrip strength—a marker of sarcopenia and global muscle wasting—serves as both a contributor to and consequence of malnutrition (47). Notably, the predictive value of handgrip strength aligns with its utility as a surrogate for overall nutritional status, reflecting both neuromuscular integrity and protein-energy reserves in stroke patients (5). Consistent with these observations, previous studies utilizing nomogram models have identified dysphagia, BI score, and grip strength as crucial risk factors for malnutrition (30, 31). Similarly, Zheng et al. identified the NIHSS scores as an independent risk factor for malnutrition in 774 stroke patients with bulbar paralysis via multiple logistic regression analysis (48). Collectively, these findings emphasize that severe stroke and functional impairment converge to amplify nutritional vulnerability, while sarcopenia mediates and exacerbates this risk.

### Clinical implications

The interpretable CAT model offers a robust framework for the proactive and personalized management of malnutrition during the subacute stage of post-stroke care. By integrating model-derived risk stratification with modifiable predictors, clinicians can effectively implement evidence-based strategies to mitigate malnutrition. Upon initial assessments, the model classifies patients into low- or high-risk categories based on predicted probabilities, thereby enabling targeted interventions for high-risk individuals characterized by factors such as advanced age, severe dysphagia, diminished handgrip strength, or dependence on tube feeding. To facilitate clinical integration, the model may be deployed as a web-based calculator, allowing clinicians to input patient data and generate immediate risk assessments. Furthermore, integrating model outputs into EHRs could trigger real-time alerts for at-risk patients, prompting timely nutritional support. These automated alerts serve to guide multidisciplinary teams—comprising neurologists, dietitians, and rehabilitation specialists—in coordinating nutritional strategies with broader recovery objectives, ensuring the effective translation of model insights into clinical practice. Targeted intervention pathways focus on optimizing modifiable predictors identified via SHAP analysis; these include resistance training and protein supplementation to enhance handgrip strength, early swallowing therapy for dysphagia management, and caloric adjustments tailored to neurological severity. Additionally, for high-risk subgroups, particularly those dependent on tube feeding or subject to prolonged immobilization, immunonutrition is recommended to counteract catabolic states and mitigate infection risks.

### Limitations

This study has several limitations that warrant consideration. First, the cross-sectional design inherently restricts causal inference, introducing potential selection bias and precluding the establishment of temporal relationships between predictors and malnutrition outcomes. Second, although the model integrates routine clinical parameters, it is essential to acknowledge the omission of critical confounders, including premorbid nutritional status indicators (e.g., pre-existing sarcopenia and frailty), psychological factors (e.g., depression severity and adequacy of caregiver support), and dynamic metabolic biomarkers (e.g., oxidative stress markers). These omissions of these variables may obscure significant modifiers of malnutrition risk and introduce residual confounding. Furthermore, due to clinical workflow constraints, gold-standard assessments for sarcopenia—specifically muscle mass and strength measurements via dual-energy X-ray absorptiometry (DEXA) and bioelectrical impedance analysis (BIA), as recommended by the GLIM criteria—were unavailable. Instead, calf circumference and handgrip strength served as surrogate measures, potentially compromising diagnostic accuracy. Consequently, these constraints obscure the delineation of the complex interplay between nutritional status and sarcopenia progression. Addressing these gaps could significantly enhance understanding of malnutrition and sarcopenia development. Third, there are ethical considerations concerning data privacy and the potential for algorithmic bias. The model relies on patient data, necessitating stringent safeguards to protect confidentiality and comply with data protection regulations. Moreover, the use of ML models raises concerns of bias if training data do not adequately represent all subpopulations, which could lead to inequities in health outcomes. Fourth, although external validation was performed, both the development and validation cohorts were recruited from hospitals affiliated with the same institutional network in the same city. This institutional overlap, combined with geographic and demographic homogeneity, restricts the generalizability of the model to broader healthcare contexts, particularly rural regions or populations with differing cultural, economic, or infrastructural characteristics that influence post-stroke recovery dynamics. Moreover, a key concern is the potential overfitting of the model due to these constraints. Overfitting may occur when the model captures noise specific to the training data rather than underlying patterns, resulting in reduced predictive performance on new unseen datasets. This risk underscores the importance of conducting additional external multicenter validation involving geographically dispersed institutions and heterogeneous patient populations to ensure the model’s robustness and validity across diverse clinical environments.

## Conclusion

This study demonstrates that an ML-based approach, specifically utilizing the CAT model, effectively predicts malnutrition risk in subacute post-stroke patients. This model not only exhibited strong predictive performance across both internal and external validations but also identified key clinical determinants of malnutrition, including advanced age, reduced handgrip strength, reliance on tube feeding, elevated NIHSS scores, diminished BI scores, and dysphagia. These findings highlight the potential of ML tools to enhance patient care by providing reliable risk stratification, thereby enabling clinicians to implement timely and targeted interventions. The integration of ML models into clinical workflows facilitates decision-making in complex scenarios, ultimately aiming to improve patient outcomes. Future work should focus on further refining these models and assessing their applicability across diverse clinical settings to enhance their generatability and clinical utility.

## Data Availability

The raw data supporting the conclusions of this article will be made available by the authors, without undue reservation.
