# Supplementary material for: Interpretable machine learning-based predictive model for malnutrition in subacute post-stroke patients: an internal and external validation study
Source: Front Nutr. 2026 Jan 5;12:1692020. doi: 10.3389/fnut.2025.1692020 (PMC12824420; doi:10.3389/fnut.2025.1692020)
Supplement: Supplementary file 1 [file Table_1.DOCX]

Supplementary Material

**Content**

**Supplementary Table S1.** Descriptions of machine learning algorithms.**2**

**Supplementary** **Table S2.** Optimal hyperparameters for the eight machine learning algorithms**3**

**Supplementary Table S3.** Baseline characteristics of stroke patients with or without malnutrition.**4**

**Supplementary Table S4.** Comparison of baseline characteristics between training and testing cohorts.**6**

**Supplementary Table S5.** Results of Delong test of the eight models.**8**

**Supplementary Table S6.** The AUC of each-fold cross validation of eight ML models.**9**

**Supplementary Table S7.** Baseline characteristics of stroke patients in the external validation cohort..**10**

**Supplementary Table S8.** Comparison of model performance in the external validation cohorts..**12**

**Supplementary Figure S1.** Comparisons of missing data before and after imputation.**13**

**Supplementary Figure S2.** Five-fold cross-validation of the training dataset.**14**

**Supplementary Table S1.** Descriptions of machine learning algorithms.

| Model | Definitions |
| --- | --- |
| Logistic Regression  (LR) | A generalized linear model that estimates class probabilities via the logistic sigmoid function, maximizing the conditional log-likelihood of observed binary outcomes. |
| Random Forest  (RF) | An ensemble of de-correlated decision trees built on bootstrap samples and random subspaces, whose aggregated majority vote (or average) yields robust predictive distributions. |
| Extreme Gradient Boosting  (XGBoost) | A scalable, regularized gradient-boosting framework that additively constructs weak learners by second-order Taylor approximation of the loss, with built-in sparsity-aware and parallelized tree splitting. |
| Light Gradient Boosting Machine (LGBM) | A high-performance GBDT variant that uses histogram-based, leaf-wise tree growth with gradient-based one-side sampling and exclusive feature bundling to reduce complexity while preserving accuracy. |
| Support Vector Machines  (SVM) | A large-margin classifier that finds the hyperplane maximizing the geometric distance to the nearest training points, implicitly mapping data via kernel functions to achieve non-linear separation. |
| Catboost  (CAT) | A non-parametric, instance-based learner that assigns labels by local majority vote among the k closest observations in the feature space, relying on distance metrics without explicit model training. |
| k-Nearest Neighbors  (KNN) | A layered composition of affine transformations followed by non-linear activations, trained by back-propagated gradient descent to approximate complex functions through distributed hidden representations. |
| Neural Network  (NNet) | An ordered boosting algorithm that combats target leakage and handles categorical variables natively via ordered target statistics, growing oblivious decision trees with feature combinations to minimize loss. |

**Supplementary** **Table S2.** Optimal hyperparameters for the eight machine learning algorithms.

| Model | Optimal parameters |
| --- | --- |
| Logistic Regression  (LR) | Alpha: 0.01, 0.25, 0.5; lambda: 0.01, 0.05, 0.1;  Family: binary |
| Random Forest  (RF) | Mtry: 2, 4; ntree：200, 300; nodesize: 35, 45;  Maxnodes: 12, 18; sampsize：200, 300; replace: TRUE  Strata: Result; importance: TRUE |
| Extreme Gradient Boosting  (XGBoost) | max_depth: 2, 3; eta: 0.02; subsample: 0.6, 0.7;  colsample_bytree: 0.6, 0.7; reg_lambda: 2.0, 2.5;  gamma: 0.2; objective: binary:logistic;  early_stopping_rounds: 30; decision threshold: 0.51 |
| Light Gradient Boosting Machine (LGBM) | num_leaves: 10, 12; reg_lambda: 3.0, 4.0;  subsample: 0.5, 0.7; min_data_in_leaf: 45;  metric: auc |
| Catboost  (CAT) | depth: 2, 3; iterations: 300, 400;  learning_rate: 0.04, 0.05; class_weights_neg: 1.2, 1.3;  reg_lambda: 1.0, 1.2; subsample: 0.9;  colsample_bylevel: 0.9; loss_function: Logloss  eval_metric: AUC |
| Support Vector Machines  (SVM) | C: 0.05, 0.1, 0.5; sigma: 0.05, 0.1, 0.5;  method: svmRadial; preProcess: center, scale;  classProbs: TRUE |
| Neural Network  (NNet) | size: 5, 7, 9; decay: 0.4, 0.5;  maxit: 350, 450; formula: Result ~ . |
| k-Nearest Neighbors  (KNN) | k: 2, 3, 5, 7, 9, 11, 13, 15, 17, 20, 25, 30;  l: 1, 2 (corresponding distance: Manhattan/Ojiri);  preProcess: zv, center, scale;  prob: TRUE |

**Supplementary** **Table S3.** Baseline characteristics of stroke patients with or without malnutrition (N = 802).

| Characteristics | Total  (N = 802) | Malnutrition  (n = 459) | Normal nutrition  (n = 343) | *P*-value^a^ |
| --- | --- | --- | --- | --- |
| Age | 64.0 [57.0, 70.0] | 66.0 [60.0, 72.0] | 62.0 [55.0, 68.0] | < 0.001 |
| Gender |  |  |  |  |
| Male | 573 (71.4%) | 323 (70.4%) | 250 (72.9%) | 0.435 |
| Female | 229 (28.6%) | 136 (29.6%) | 93 (27.1%) |  |
| BMI (kg/m^2^) | 25.1 [22.3, 28.5] | 25.0 [22.0, 28.6] | 25.4 [22.9, 28.4] | 0.030 |
| Medical fee payment |  |  |  |  |
| Self-pay | 112 (14.0%) | 63 (13.7%) | 49 (14.3%) | 0.821 |
| Insurance | 690 (86.0%) | 396 (86.3%) | 294 (85.7%) |  |
| Education level |  |  |  |  |
| <high school | 513 (64.0%) | 287 (62.5%) | 226 (65.9%) | 0.327 |
| ≥high school | 289 (36.0%) | 172 (37.5%) | 117 (34.1%) |  |
| Employment status |  |  |  |  |
| Employed | 188 (23.4%) | 104 (22.7%) | 84 (24.5%) | 0.545 |
| Unemployed/retired | 614 (76.6%) | 355 (77.3%) | 259 (75.5%) |  |
| Living along | 101 (12.6%) | 53 (11.5%) | 48 (14.0%) | 0.301 |
| Monthly family income (RMB) | |  |  |  |
| < 2000 | 164 (20.4%) | 90 (19.6%) | 74 (21.6%) | 0.789 |
| 2000-5000 | 511 (63.7%) | 296 (64.5%) | 215 (62.7%) |  |
| > 5000 | 127 (15.8%) | 73 (15.9%) | 54 (15.7%) |  |
| Drinking history | 364 (45.4%) | 219 (47.7%) | 145 (42.3%) | 0.126 |
| Smoking history | 317 (39.5%) | 195 (42.5%) | 122 (35.6%) | 0.048 |
| Hypertension | 360 (44.9%) | 195 (42.5%) | 165 (48.1%) | 0.113 |
| Diabetes | 233 (29.1%) | 139 (30.3%) | 94 (27.4%) | 0.374 |
| Dyslipidemia | 277 (34.5%) | 162 (35.3%) | 115 (33.5%) | 0.603 |
| Cardiovascular disease | 422 (52.6%) | 244 (53.2%) | 178 (51.9%) | 0.723 |
| Digestive disease | 133 (16.6%) | 83 (18.1%) | 50 (14.6%) | 0.187 |
| Chronic kidney disease | 67 (8.4%) | 35 (7.6%) | 32 (9.3%) | 0.388 |
| Eating habits |  |  |  |  |
| Oral | 708 (88.3%) | 387 (84.3%) | 321 (93.6%) | < 0.001 |
| Tube feeding | 94 (11.7%) | 72 (15.7%) | 22 (6.4%) |  |
| Polypharmacy (>5) | 241 (30.0%) | 127 (27.7%) | 114 (33.2%) | 0.089 |
| BI score | 65.0 [50.0, 75.0] | 60.0 [50.0, 75.0] | 65.0 [55.0, 80.0] | < 0.001 |
| Handgrip strength (kg) | 21.0 [17.0, 25.0] | 20.0 [16.0, 24.0] | 22.0 [17.0, 27.0] | < 0.001 |
| Days from stroke onset | 38.0 [29.0, 46.0] | 37.0 [29.0, 46.0] | 38.0 [29.0, 47.0] | 0.999 |
| Stroke type |  |  |  |  |
| Ischemic | 694 (86.5%) | 396 (86.3%) | 298 (86.9%) | 0.804 |
| Hemorrhagic | 108 (13.5%) | 63 (13.7%) | 45 (13.1%) |  |
| Lesion sides |  |  |  |  |
| Left | 391 (48.8%) | 221 (48.1%) | 170 (49.6%) | 0.750 |
| Right | 271 (33.8%) | 160 (34.9%) | 111 (32.4%) |  |
| Bilaterial | 140 (17.5%) | 78 (17.0%) | 62 (18.1%) |  |
| NIHSS score | 6.0 [5.0, 9.0] | 7.0 [5.0, 10.0] | 6.0 [4.0, 7.0] | < 0.001 |
| Post-stroke complications |  |  |  |  |
| Pneumonia | 70 (8.7%) | 44 (9.6%) | 26 (7.6%) | 0.319 |
| Paresis at dominant arm | 257 (32.0%) | 152 (33.1%) | 105 (30.6%) | 0.452 |
| Loss of appetite | 159 (19.8%) | 103 (22.4%) | 56 (16.3%) | 0.032 |
| Dysphagia | 335 (41.8%) | 224 (48.8%) | 111 (32.4%) | <0.001 |
| Dysarthria/aphasia | 471 (58.7%) | 268 (58.4%) | 203 (59.2%) | 0.821 |
| Laboratory test |  |  |  |  |
| HGB (g/L) | 129.0 [113.0, 145.0] | 129.0 [113.0, 145.0] | 130.0 [114.0, 145.0] | 0.722 |
| TP (g/L) | 65.0 [57.0, 73.0] | 65.0 [57.0, 73.0] | 65.0 [58.0, 73.0] | 0.654 |
| TC (mmol/L) | 4.6 [3.2, 6.0] | 4.5 [3.2, 6.0] | 4.7 [3.1, 6.0] | 0.968 |
| TG (mmol/L) | 1.2 [0.7, 1.7] | 1.2 [0.8, 1.7] | 1.2 [0.7, 1.7] | 0.307 |
| Scr (mmol/L) | 116.0 [72.0, 162.0] | 113.0 [71.0, 162.0] | 121.0 [74.0, 162.0] | 0.275 |
| FIB (g/L) | 4.5 [3.1, 6.2] | 4.7 [3.2, 6.3] | 4.4 [2.9, 5.9] | 0.006 |
| D-dimer (μg/mL) | 1.1 [0.8, 1.4] | 1.1 [0.8, 1.4] | 1.1 [0.8, 1.4] | 0.722 |
| HDL-C (mmol/L) | 1.4 [0.8, 1.9] | 1.4 [0.8, 2.0] | 1.4 [0.8, 1.9] | 0.319 |
| LDL-C (mmol/L) | 2.3 [1.6, 3.1] | 2.3 [1.6, 3.1] | 2.3 [1.5, 3.2] | 0.969 |
| CRP (mg/L) | 16.0 [10.0, 22.0] | 16.0 [10.0, 23.0] | 15.0 [9.0, 21.0] | 0.025 |
| UA (μmol/L) | 313.0 [244.8, 386.3] | 310.0 [242.0, 386.0] | 319.0 [245.0, 388.0] | 0.478 |
| WBC (10^9^/L) | 10.5 [6.7, 13.8] | 10.5 [6.9, 13.8] | 10.3 [6.4, 13.7] | 0.502 |
| NEU (10^9^/L) | 4.3 [3.4, 5.6] | 4.4 [3.4, 5.6] | 4.3 [3.4, 5.6] | 0.687 |
| LYM (10^9^/L) | 1.6 [1.1, 2.2] | 1.5 [1.1, 2.1] | 1.6 [1.1, 2.2] | 0.929 |
| PLA (10^9^/L) | 225.0 [172.0, 292.0] | 226.0 [174.0, 293.0] | 223.0 [171.0, 290.0] | 0.957 |
| Alb (g/L) | 36.0 [30.0, 43.0] | 35.0 [29.0, 42.0] | 37.0 [31.0, 43.0] | 0.067 |
| NLR | 2.8 [1.8, 4.2] | 2.9 [1.9, 4.4] | 2.7 [1.8, 4.1] | 0.969 |
| PLR | 143.3 [94.2, 212.8] | 142.7 [93.6, 223.3] | 143.8 [94.6, 202.0] | 0.847 |
| PNI | 45.5 [38.5, 51.5] | 44.5 [37.5, 50.5] | 46.5 [39.5, 52.5] | 0.101 |

Notes. ^a^Categorical variables were compared with Chi-square test, while the continuous variables were compared with Mann-Whitney U test.

Abbreviations: BMI, body mass index; BI: Barthel Index, NIHSS, National Institutes of Health Stroke Scale; HGB, hemoglobin; TP, total protein; TC, total cholesterol; TG, triglycerides; Scr, serum creatinine; FIB, fibrinogen; HDL-C, high-density lipoprotein cholesterol; LDL-C, low-density lipoprotein cholesterol; CRF, C-reaction protein; UA, uric acid; WBC, while blood cell; NEU, neutrophils; LYM, lymphocytes; PLA, platelets; Alb, albumin; NLR, neutrophil-lymphocyte ratio; PLR, platelet-to-lymphocyte ratio; PNI, prognostic nutritional index.

*P* < 0.05, statistically significant.

**Supplementary Table S4.** Comparison of baseline characteristics between training and testing cohorts.

| Characteristics | Total  (N = 802) | Training cohort  (N = 562) | Testing cohort  (N = 240) | *P*-value^a^ |
| --- | --- | --- | --- | --- |
| Age | 64.0 [57.0, 70.0] | 64.0 [57.0, 70.0] | 64.0 [58.0, 70.0] | 0.426 |
| Gender |  |  |  |  |
| Male | 573 (71.4%) | 409 (72.8%) | 164 (68.3%) | 0.202 |
| Female | 229 (28.6%) | 153 (27.2%) | 76 (31.7%) |  |
| BMI (kg/m^2^) | 25.1 [22.3, 28.5] | 25.0 [22.4, 28.5] | 25.3 [22.3, 28.5] | 0.996 |
| Medical fee payment |  |  |  |  |
| Self-pay | 112 (14.0%) | 75 (13.3%) | 37 (15.4%) | 0.438 |
| Insurance | 690 (86.0%) | 487 (86.7%) | 203 (84.6%) |  |
| Education level |  |  |  |  |
| <high school | 513 (64.0%) | 364 (64.8%) | 149 (62.1%) | 0.468 |
| ≥high school | 289 (36.0%) | 198 (35.2%) | 91 (37.9%) |  |
| Employment status |  |  |  |  |
| Employed | 188 (23.4%) | 125 (22.2%) | 63 (26.3%) | 0.220 |
| Unemployed/retired | 614 (76.6%) | 437 (77.8%) | 177 (73.8%) |  |
| Living along | 101 (12.6%) | 68 (12.1%) | 33 (13.8%) | 0.519 |
| Monthly family income (RMB) | |  |  |  |
| < 2000 | 164 (20.4%) | 116 (20.6%) | 48 (20.0%) | 0.768 |
| 2000-5000 | 511 (63.7%) | 354 (63.0%) | 157 (65.4%) |  |
| > 5000 | 127 (15.8%) | 92 (16.4%) | 35 (14.6%) |  |
| Drinking history | 364 (45.4%) | 243 (43.2%) | 121 (50.4%) | 0.062 |
| Smoking history | 317 (39.5%) | 223 (39.7%) | 94 (39.2%) | 0.892 |
| Hypertension | 360 (44.9%) | 255 (45.4%) | 105 (43.8%) | 0.672 |
| Diabetes | 233 (29.1%) | 156 (27.8%) | 77 (32.1%) | 0.217 |
| Dyslipidemia | 277 (34.5%) | 196 (34.9%) | 81 (33.8%) | 0.759 |
| Cardiovascular disease | 422 (52.6%) | 305 (54.3%) | 117 (48.8%) | 0.152 |
| Digestive disease | 133 (16.6%) | 89 (15.8%) | 44 (18.3%) | 0.384 |
| Chronic kidney disease | 67 (8.4%) | 49 (8.7%) | 18 (7.5%) | 0.568 |
| Eating habits |  |  |  |  |
| Oral | 708 (88.3%) | 503 (89.5%) | 205 (85.4%) | 0.100 |
| Tube feeding | 94 (11.7%) | 59 (10.5%) | 35 (14.6%) |  |
| Polypharmacy (>5) | 241 (30.0%) | 169 (30.1%) | 72 (30.0%) | 0.984 |
| BI score | 65.0 [50.0, 75.0] | 65.0 [50.0, 75.0] | 65.0 [55.0, 75.0] | 0.451 |
| Handgrip strength (kg) | 21.0 [17.0, 25.0] | 21.0 [16.0, 25.0] | 20.5 [17.0, 25.0] | 0.895 |
| Days from stroke onset | 38.0 [29.0, 46.0] | 38.0 [29.0, 46.0] | 38.0 [29.0, 46.0] | 0.664 |
| Stroke type |  |  |  |  |
| Ischemic | 694 (86.5%) | 489 (87.0%) | 205 (85.4%) | 0.545 |
| Hemorrhagic | 108 (13.5%) | 73 (13.0%) | 35 (14.6%) |  |
| Lesion sides |  |  |  |  |
| Left | 391 (48.8%) | 274 (48.8%) | 117 (48.8%) | 0.780 |
| Right | 271 (33.8%) | 193 (34.3%) | 78 (32.5%) |  |
| Bilaterial | 140 (17.5%) | 95 (16.9%) | 45 (18.8%) |  |
| NIHSS score | 6.0 [5.0, 9.0] | 6.0 [5.0, 9.0] | 7.0 [5.0, 9.0] | 0.268 |
| Post-stroke complications |  |  |  |  |
| Pneumonia | 70 (8.7%) | 52 (9.3%) | 18 (7.5%) | 0.421 |
| Paresis at dominant arm | 257 (32.0%) | 175 (31.1%) | 82 (34.2%) | 0.400 |
| Loss of appetite | 159 (19.8%) | 117 (20.8%) | 42 (17.5%) | 0.280 |
| Dysphagia | 335 (41.8%) | 235 (41.8%) | 100 (41.7%) | 0.969 |
| Dysarthria/aphasia | 471 (58.7%) | 327 (58.2%) | 144 (60.0%) | 0.633 |
| Laboratory test |  |  |  |  |
| HGB (g/L) | 129.0 [113.0, 145.0] | 129.0 [113.0, 144.0] | 131.0 [114.0, 147.8] | 0.597 |
| TP (g/L) | 65.0 [57.0, 73.0] | 66.0 [58.0, 74.0] | 65.0 [57.0, 71.8] | 0.105 |
| TC (mmol/L) | 4.6 [3.2, 6.0] | 4.6 [3.1, 6.0] | 4.5 [3.2, 5.9] | 0.867 |
| TG (mmol/L) | 1.2 [0.7, 1.7] | 1.3 [0.8, 1.7] | 1.2 [0.7, 1.7] | 0.098 |
| Scr (mmol/L) | 116.0 [72.0, 162.0] | 112.0 [71.0, 159.0] | 124.0 [75.5, 169.0] | 0.072 |
| FIB (g/L) | 4.5 [3.1, 6.2] | 4.6 [3.2, 6.2] | 4.3 [2.9, 6.1] | 0.068 |
| D-dimer (μg/mL) | 1.1 [0.8, 1.4] | 1.1 [0.8, 1.4] | 1.1 [0.8, 1.4] | 0.410 |
| HDL-C (mmol/L) | 1.4 [0.8, 1.9] | 1.5 [0.8, 1.9] | 1.4 [0.8, 2.0] | 0.919 |
| LDL-C (mmol/L) | 2.3 [1.6, 3.1] | 2.4 [1.6, 3.2] | 2.3 [1.5, 3.1] | 0.596 |
| CRP (mg/L) | 16.0 [10.0, 22.0] | 16.0 [10.0, 22.0] | 15.5 [10.0, 22.0] | 0.876 |
| UA (μmol/L) | 313.0 [244.8, 386.3] | 312.0 [239.8, 386.0] | 316.0 [254.0, 393.3] | 0.382 |
| WBC (10^9^/L) | 10.5 [6.7, 13.8] | 10.5 [6.7, 13.8] | 10.5 [6.8, 13.7] | 0.995 |
| NEU (10^9^/L) | 4.3 [3.4, 5.6] | 4.5 [3.4, 5.7] | 4.3 [3.3, 5.2] | 0.090 |
| LYM (10^9^/L) | 1.6 [1.1, 2.2] | 1.6 [1.1, 2.2] | 1.7 [1.1, 2.2] | 0.725 |
| PLA (10^9^/L) | 225.0 [172.0, 292.0] | 226.5 [174.8, 292.0] | 220.0 [163.5, 294.0] | 0.736 |
| Alb (g/L) | 36.0 [30.0, 43.0] | 36.0 [29.0, 43.0] | 37.0 [31.0, 42.8] | 0.611 |
| NLR | 2.8 [1.8, 4.2] | 2.9 [1.8, 4.3] | 2.5 [1.9, 4.0] | 0.262 |
| PLR | 143.3 [94.2, 212.8] | 144.6 [94.6, 218.5] | 142.3 [92.4, 201.5] | 0.567 |
| PNI | 45.5 [38.5, 51.5] | 45.3 [38.0, 51.5] | 45.5 [39.0, 51.0] | 0.922 |
| Malnutrition |  |  |  |  |
| No | 343 (42.8%) | 248 (44.1%) | 95 (39.6%) | 0.234 |
| Yes | 459 (57.2%) | 314 (55.9%) | 145 (60.4%) |  |

**Notes.** ^a^Categorical variables were compared with Chi-square test, while the continuous variables were compared with Mann-Whitney U test.

**Abbreviations:** BMI, body mass index; BI: Barthel Index, NIHSS, National Institutes of Health Stroke Scale; HGB, hemoglobin; TP, total protein; TC, total cholesterol; TG, triglycerides; Scr, serum creatinine; FIB, fibrinogen; HDL-C, high-density lipoprotein cholesterol; LDL-C, low-density lipoprotein cholesterol; CRP, C-reaction protein; UA, uric acid; WBC, while blood cell; NEU, neutrophils; LYM, lymphocytes; PLA, platelets; Alb, albumin; NLR, neutrophil-lymphocyte ratio; PLR, platelet-to-lymphocyte ratio; PNI, prognostic nutritional index.

*P* < 0.05, statistically significant.

**Supplementary Table S5.** Results of Delong test of the eight models.

| Training set | Comparison Model | AUC Difference | Z Value | *P* Value |
| --- | --- | --- | --- | --- |
|  | CAT vs LR | 0.080 | 7.503 | <0.001 |
|  | CAT vs RF | 0.050 | 6.192 | <0.001 |
|  | CAT vs XGBoost | 0.027 | 5.527 | <0.001 |
|  | CAT vs LGBM | 0.122 | 7.936 | <0.001 |
|  | CAT vs SVM | 0.082 | 7.715 | <0.001 |
|  | CAT vs NNet | 0.052 | 5.703 | <0.001 |
|  | CAT vs KNN | 0.086 | 6.851 | <0.001 |
| Testing set | Comparison Model | AUC Difference | Z Value | *P* Value |
|  | CAT vs LR | 0.032 | 2.289 | 0.022 |
|  | CAT vs RF | 0.050 | 3.724 | <0.001 |
|  | CAT vs XGBoost | 0.023 | 2.711 | 0.007 |
|  | CAT vs LGBM | 0.074 | 3.499 | <0.001 |
|  | CAT vs SVM | 0.033 | 2.271 | 0.023 |
|  | CAT vs NNet | 0.034 | 2.434 | 0.015 |
|  | CAT vs KNN | 0.092 | 4.253 | 0.000 |

**Note. Abbreviations:** AUC: the area under the receiver operating characteristic ROC curve; LR, Logistic Regression; RF, Random Forests; XGBoost, Extreme Gradient Boosting; LGBM, Light Gradient Boosting Machine; SVM, Support Vector Machines; KNN, k-Nearest Neighbors; NNet, Neural Network; CAT, CatBoost.

**Supplementary Table S6.** The AUC of each-fold cross validation of eight ML models.

| Model | Fold_1 | Fold_2 | Fold_3 | Fold_4 | Fold_5 | AUC (mean ± SD) |
| --- | --- | --- | --- | --- | --- | --- |
| CAT | 0.721 (0.619-0.823) | 0.796 (0.716-0.877) | 0.827 (0.750-0.904) | 0.742 (0.652-0.833) | 0.729 (0.635-0.823) | 0.763 ± 0.046 (0.706-0.820) |
| LR | 0.705 (0.601-0.808) | 0.789 (0.706-0.873) | 0.788 (0.701-0.876) | 0.805 (0.725-0.886) | 0.704 (0.608-0.800) | 0.758 ± 0.049 (0.697-0.820) |
| RF | 0.692 (0.585-0.800) | 0.756 (0.665-0.847) | 0.772 (0.680-0.864) | 0.724 (0.630-0.819) | 0.641 (0.539-0.744) | 0.717 ± 0.052 (0.652-0.782) |
| XGB | 0.701 (0.597-0.806) | 0.754 (0.664-0.843) | 0.768 (0.677-0.859) | 0.715 (0.621-0.808) | 0.727 (0.633-0.820) | 0.733 ± 0.028 (0.699-0.767) |
| LGBM | 0.64 (0.531-0.749) | 0.708 (0.611-0.805) | 0.746 (0.650-0.843) | 0.652 (0.551-0.753) | 0.684 (0.584-0.784) | 0.686 ± 0.043 (0.633-0.739) |
| SVM | 0.691 (0.587-0.796) | 0.77 (0.681-0.859) | 0.779 (0.689-0.870) | 0.78 (0.695-0.864) | 0.668 (0.568-0.768) | 0.738 ± 0.054 (0.671-0.804) |
| NNet | 0.674 (0.568-0.780) | 0.76 (0.671-0.850) | 0.75 (0.656-0.843) | 0.727 (0.633-0.821) | 0.674 (0.575-0.774) | 0.717 ± 0.041 (0.666-0.768) |
| KNN | 0.628 (0.521-0.734) | 0.749 (0.659-0.840) | 0.714 (0.614-0.814) | 0.645 (0.546-0.745) | 0.629 (0.528-0.730) | 0.673 ± 0.055 (0.604-0.742) |

**Note. Abbreviations:** AUC: the area under the receiver operating characteristic ROC curve; LR, Logistic Regression; RF, Random Forests; XGBoost, Extreme Gradient Boosting; LGBM, Light Gradient Boosting Machine; SVM, Support Vector Machines; KNN, k-Nearest Neighbors; NNet, Neural Network; CAT, CatBoost.

**Supplementary Table S7.** Baseline characteristics of stroke patients in the external validation cohort.

| Characteristics | Total  (N = 345) | Malnutrition  (N = 188) | Normal nutrition  (N = 155) | Statistics^a^ | p-value |
| --- | --- | --- | --- | --- | --- |
| Age | 65.0 [57.0, 71.0] | 66.0 [60.0, 73.0] | 61.0 [55.0, 68.0] | 4.518 | < 0.001 |
| Gender |  |  |  |  |  |
| Male | 240 (70.0%) | 135 (71.8%) | 105 (67.7%) | 0.669 | 0.414 |
| Female | 103 (30.0%) | 53 (28.2%) | 50 (32.3%) |  |  |
| BMI (kg/m^2^) | 24.9 [22.1, 28.3] | 24.5 [21.4, 28.4] | 25.1 [22.6, 28.3] | 1.597 | 0.110 |
| Medical fee payment |  |  |  |  |  |
| Self-pay | 61 (17.8%) | 39 (20.7%) | 22 (14.2%) | 2.494 | 0.114 |
| Insurance | 282 (82.2%) | 149 (79.3%) | 133 (85.8%) |  |  |
| Education level |  |  |  |  |  |
| <high school | 233 (67.9%) | 133 (70.7%) | 100 (64.5%) | 1.513 | 0.219 |
| ≥high school | 110 (32.1%) | 55 (29.3%) | 55 (35.5%) |  |  |
| Employment status |  |  |  |  |  |
| Employed | 69 (20.1%) | 39 (20.7%) | 30 (19.4%) | 0.102 | 0.749 |
| Unemployed/retired | 274 (79.9%) | 149 (79.3%) | 125 (80.6%) |  |  |
| Living along | 26 (7.6%) | 8 (4.3%) | 18 (11.6%) | 6.565 | 0.010 |
| Monthly family income (RMB) | |  |  |  |  |
| < 2000 | 70 (20.4%) | 35 (18.6%) | 35 (22.6%) | 0.830 | 0.660 |
| 2000-5000 | 220 (64.1%) | 123 (65.4%) | 97 (62.6%) |  |  |
| > 5000 | 53 (15.5%) | 30 (16.0%) | 23 (14.8%) |  |  |
| Drinking history | 150 (43.7%) | 89 (47.3%) | 61 (39.4%) | 2.202 | 0.138 |
| Smoking history | 131 (38.2%) | 63 (33.5%) | 68 (43.9%) | 3.863 | 0.049 |
| Hypertension | 162 (47.2%) | 98 (52.1%) | 64 (41.3%) | 4.003 | 0.045 |
| Diabetes | 79 (23.0%) | 53 (28.2%) | 26 (16.8%) | 6.247 | 0.012 |
| Dyslipidemia | 126 (36.7%) | 66 (35.1%) | 60 (38.7%) | 0.475 | 0.491 |
| Cardiovascular disease | 172 (50.1%) | 97 (51.6%) | 75 (48.4%) | 0.350 | 0.554 |
| Digestive disease | 51 (14.9%) | 30 (16%) | 21 (13.5%) | 0.390 | 0.533 |
| Chronic kidney disease | 35 (10.2%) | 22 (11.7%) | 13 (8.4%) | 1.019 | 0.313 |
| Eating habits |  |  |  |  |  |
| Oral | 311 (90.7%) | 166 (88.3%) | 145 (93.5%) | 2.769 | 0.096 |
| Tube feeding | 32 (9.3%) | 22 (11.7%) | 10 (6.5%) |  |  |
| Polypharmacy (>5) | 91 (26.5%) | 57 (30.3%) | 34 (21.9%) | 3.063 | 0.080 |
| BI score | 65.0 [50.0, 75.0] | 60.0 [50.0, 70.0] | 70.0 [55.0, 80.0] | -4.745 | < 0.001 |
| Handgrip strength (kg) | 20.0 [16.0, 24.0] | 20.5 [16.0, 24.0] | 20.0 [18.0, 25.0] | 1.644 | 0.100 |
| Days from stroke onset | 37.0 [27.0, 45.0] | 37.0 [26.0, 45.0] | 35.0 [28.0, 45.0] | 0.547 | 0.584 |
| Stroke type |  |  |  |  |  |
| Ischemic | 290 (84.5%) | 159 (84.6%) | 131 (84.5%) | 0.000 | 0.988 |
| Hemorrhagic | 53 (15.5%) | 29 (15.4%) | 24 (15.5%) |  |  |
| Lesion sides |  |  |  |  |  |
| Left | 159 (46.4%) | 89 (47.3%) | 70 (45.2%) | 0.428 | 0.807 |
| Right | 129 (37.6%) | 71 (37.8%) | 58 (37.4%) |  |  |
| Bilaterial | 55 (16.0%) | 28 (14.9%) | 27 (17.4%) |  |  |
| NIHSS score | 6.0 [5.0, 9.0] | 7.0 [5.0, 10.0] | 6.0 [5.0, 8.0] | 3.063 | 0.002 |
| Post-stroke complications |  |  |  |  |  |
| Pneumonia | 32 (9.3%) | 15 (8.0%) | 17 (11.0%) | 0.897 | 0.344 |
| Paresis at dominant arm | 123 (35.9%) | 62 (33.0%) | 61 (39.4%) | 1.502 | 0.220 |
| Loss of appetite | 82 (23.9%) | 52 (27.7%) | 30 (19.4%) | 3.221 | 0.073 |
| Dysphagia | 136 (39.7%) | 90 (47.9%) | 46 (29.7%) | 11.754 | < 0.001 |
| Dysarthria/aphasia | 193 (56.3%) | 110 (58.5%) | 83 (53.5%) | 0.850 | 0.357 |
| Laboratory test |  |  |  |  |  |
| HGB (g/L) | 130.0 [114.0, 145.0] | 129.0 [113.0, 145.0] | 130.0 [115.0, 143.0] | 0.021 | 0.983 |
| TP (g/L) | 64.0 [56.0, 73.0] | 63.0 [56.0, 72.8] | 65.0 [57.0, 73.0] | -0.844 | 0.399 |
| TC (mmol/L) | 4.5 [3.0, 5.9] | 4.4 [3.0, 5.7] | 4.6 [2.8, 6.0] | -0.379 | 0.705 |
| TG (mmol/L) | 1.2 [0.7, 1.7] | 1.2 [0.8, 1.8] | 1.3 [0.6, 1.6] | -0.406 | 0.684 |
| Scr (mmol/L) | 119.0 [75.0, 158.0] | 120.5 [75.0, 158.8] | 116.0 [76.0, 158.0] | -0.053 | 0.958 |
| FIB (g/L) | 4.6 [3.0, 6.3] | 4.9 [3.0, 6.5] | 4.3 [2.9, 6.1] | -1.518 | 0.129 |
| D-dimer (μg/mL) | 1.2 [0.8, 1.4] | 1.2 [0.9, 1.5] | 1.2 [0.8, 1.4] | -0.892 | 0.373 |
| HDL-C (mmol/L) | 1.4 [0.8, 2.0] | 1.4 [0.8, 2.0] | 1.4 [0.9, 1.9] | -0.440 | 0.660 |
| LDL-C (mmol/L) | 2.3 [1.6, 3.0] | 2.4 [1.6, 2.9] | 2.2 [1.7, 3.2] | -0.814 | 0.416 |
| CRP (mg/L) | 16.0 [10.0, 22.0] | 16.0 [10.0, 23.0] | 15.0 [10.0, 20.0] | -1.016 | 0.310 |
| UA (μmol/L) | 307.0 [225.0, 390.0] | 314.5 [232.5, 397.5] | 295.0 [211.0, 377.0] | -1.640 | 0.101 |
| WBC (109/L) | 10.7 [7.3, 14.1] | 10.7 [7.4, 14.0] | 10.8 [7.3, 14.3] | -0.255 | 0.798 |
| NEU (109/L) | 4.2 [3.3, 5.7] | 4.4 [3.3, 5.8] | 4.0 [3.2, 5.2] | -2.111 | 0.035 |
| LYM (109/L) | 1.7 [1.2, 2.2] | 1.7 [1.2, 2.2] | 1.6 [1.1, 2.2] | -0.423 | 0.672 |
| PLAT (109/L) | 234.0 [172.0, 299.0] | 236.5 [175.0, 290.0] | 230.0 [170.0, 306.0] | -0.095 | 0.924 |
| Alb (g/L) | 35.0 [30.0, 43.0] | 35.5 [30.0, 43.8] | 35.0 [30.0, 43.0] | -0.589 | 0.556 |
| NLR | 2.6 [1.8, 3.7] | 2.7 [1.8, 3.9] | 2.6 [1.8, 3.6] | -1.007 | 0.314 |
| PLR | 141.1 [95.4, 202.7] | 138.6 [94.3, 210.8] | 143.3 [96.8, 202.4] | -0.268 | 0.789 |
| PNI | 45.0 [38.0, 52.5] | 45.3 [39.0, 53.0] | 44.5 [36.5, 51.0] | -0.566 | 0.572 |

**Notes.** ^a^Categorical variables were compared with Chi-square test, while the continuous variables were compared with Mann-Whitney U test.

**Abbreviations:** BMI, body mass index; BI: Barthel Index, NIHSS, National Institutes of Health Stroke Scale; HGB, hemoglobin; TP, total protein; TC, total cholesterol; TG, triglycerides; Scr, serum creatinine; FIB, fibrinogen; HDL-C, high-density lipoprotein cholesterol; LDL-C, low-density lipoprotein cholesterol; CRP, C-reaction protein; UA, uric acid; WBC, while blood cell; NEU, neutrophils; LYM, lymphocytes; PLA, platelets; Alb, albumin; NLR, neutrophil-lymphocyte ratio; PLR, platelet-to-lymphocyte ratio; PNI, prognostic nutritional index.

*P* < 0.05, statistically significant.

**Supplementary Table S8.** Comparison of model performance in the external validation cohorts.

| Model | Accuracy (95% CI) | AUC (95% CI) | Recall (95% CI) | Precision (95% CI) | F1 score (95% CI) | Specificity (95% CI) | Log loss (95% CI) | Brier score (95% CI) |
| --- | --- | --- | --- | --- | --- | --- | --- | --- |
| LR | 0.685 (0.636-0.735) | 0.745 (0.693-0.796) | 0.750 (0.690-0.814) | 0.698 (0.630-0.758) | 0.723 (0.668-0.771) | 0.606 (0.524-0.685) | 0.586 (0.542-0.632) | 0.202 (0.182-0.222) |
| RF | 0.685 (0.635-0.732) | 0.744 (0.691-0.796) | 0.766 (0.706-0.823) | 0.692 (0.626-0.755) | 0.727 (0.675-0.774) | 0.587 (0.510-0.665) | 0.603 (0.545-0.657) | 0.206 (0.183-0.228) |
| XGBoost | 0.697 (0.647-0.749) | 0.763 (0.713-0.813) | 0.723 (0.672-0.797) | 0.723 (0.654-0.784) | 0.723 (0.676-0.780) | 0.665 (0.578-0.729) | 0.582 (0.523-0.642) | 0.200 (0.175-0.225) |
| LGBM | 0.650 (0.601-0.700) | 0.700 (0.644-0.755) | 0.745 (0.684-0.804) | 0.660 (0.595-0.726) | 0.700 (0.645-0.748) | 0.535 (0.459-0.614) | 0.628 (0.593-0.664) | 0.219 (0.203-0.235) |
| CAT | 0.700 (0.650-0.749) | 0.772 (0.723-0.820) | 0.718 (0.646-0.778) | 0.730 (0.662-0.795) | 0.724 (0.667-0.772) | 0.677 (0.601-0.756) | 0.560 (0.519-0.603) | 0.193 (0.175-0.211) |
| SVM | 0.682 (0.633-0.729) | 0.738 (0.685-0.790) | 0.723 (0.659-0.787) | 0.705 (0.637-0.766) | 0.714 (0.659-0.762) | 0.632 (0.558-0.706) | 0.599 (0.555-0.649) | 0.207 (0.187-0.229) |
| NNet | 0.703 (0.650-0.746) | 0.753 (0.702-0.804) | 0.750 (0.691-0.813) | 0.719 (0.660-0.784) | 0.734 (0.692-0.780) | 0.645 (0.567-0.723) | 0.581 (0.534-0.628) | 0.199 (0.178-0.219) |
| KNN | 0.671 (0.618-0.717) | 0.721 (0.668-0.775) | 0.782 (0.722-0.837) | 0.671 (0.610-0.729) | 0.722 (0.672-0.765) | 0.535 (0.456-0.611) | 0.612 (0.573-0.652) | 0.212 (0.194-0.229) |

**Note. Abbreviations:** AUC: the area under the receiver operating characteristic ROC curve; LR, Logistic Regression; RF, Random Forests; XGBoost, Extreme Gradient Boosting; LGBM, Light Gradient Boosting Machine; SVM, Support Vector Machines; KNN, k-Nearest Neighbors; NNet, Neural Network; CAT, CatBoost.

**Supplementary Figure S1.**


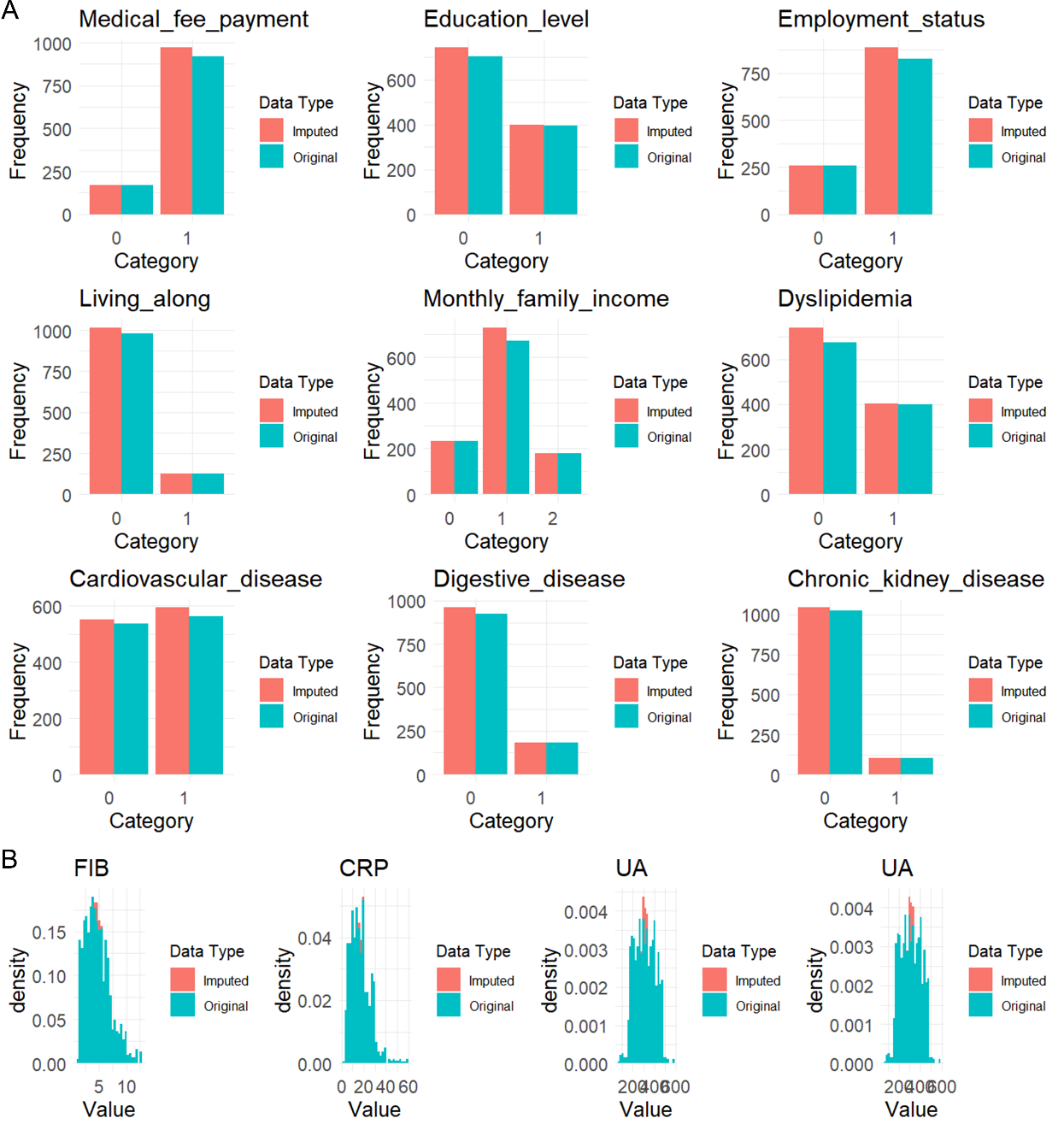


**Supplementary Figure S1.** Comparisons of missing data before and after imputation. Abbreviations: FIB, fibrinogen; CRP, C-reaction protein; UA, uric acid; TP, total protein.

**Supplementary Figure S2.**


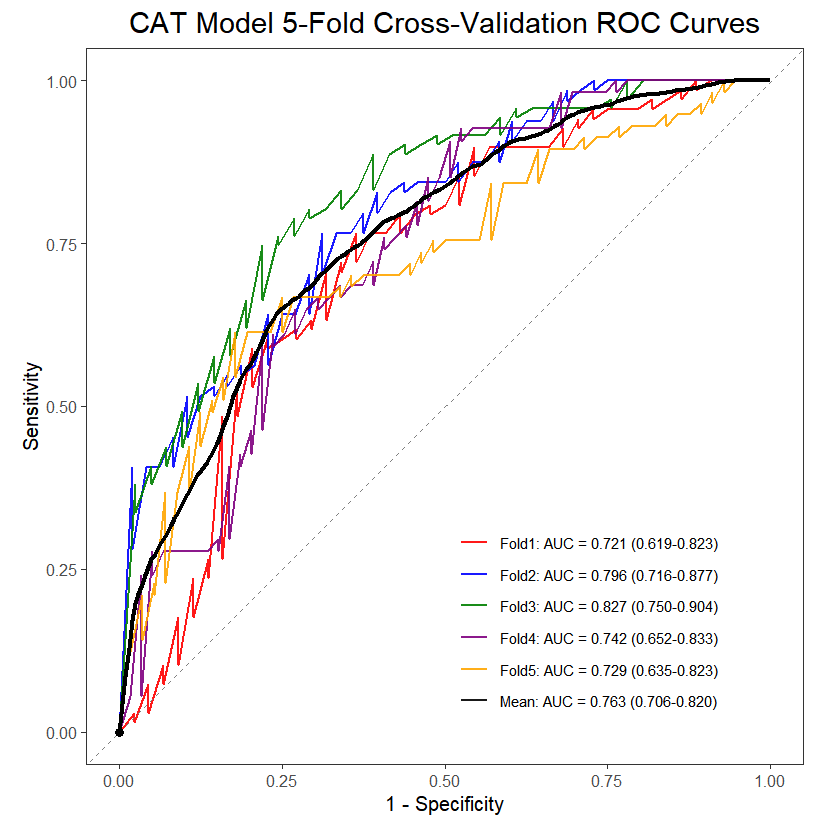


**Supplementary Figure S2.** Five-fold cross-validation of the training dataset.
